# The endTB observational study protocol: treatment of MDR-TB with bedaquiline or delamanid containing regimens

**DOI:** 10.1186/s12879-019-4378-4

**Published:** 2019-08-20

**Authors:** Uzma Khan, Helena Huerga, Aamir J. Khan, Carole D. Mitnick, Catherine Hewison, Francis Varaine, Mathieu Bastard, Michael Rich, Molly F. Franke, Sidney Atwood, Palwasha Y. Khan, Kwonjune J. Seung

**Affiliations:** 1 Interactive Research and Development (IRD), Dubai, United Arab Emirates; 2 Field Epidemiology Department, Epicentre, Paris, France; 3 Interactive Research and Development (IRD) Global, Singapore, Singapore; 4 Department of Global Health and Social Medicine, Harvard Medical School, Boston, USA; 5 Medical Department, Médecins Sans Frontières, Paris, France; 6 Partners In Health, Boston, USA; 7 Division of Global Health Equity, Brigham and Women’s Hospital, Boston, USA

**Keywords:** MDR-TB, Bedaquiline, Delamanid, Safety, Efficacy

## Abstract

**Background:**

At a time when programs were struggling to design effective regimens for the treatment of multidrug-resistant tuberculosis (MDR-TB), the marketing authorization of bedaquiline and delamanid was a critical development in the MDR-TB treatment landscape. However, despite their availability for routine programmatic use, the uptake of these drugs has remained slow; concerns included a lack of evidence on safety and efficacy and the need to protect the new drugs from the development of acquired resistance. As part of the endTB Project, we aimed to address these barriers by generating evidence on safety and efficacy of bedaquiline or delamanid based MDR-TB regimens.

**Methods:**

This is a protocol for a multi-center prospective cohort study to enroll 2600 patients from April 2015 through September 2018 in 17 countries. The protocol describes inclusion of patients started on treatment with bedaquiline- or delamanid- containing regimens under routine care, who consented to participate in the endTB observational study. Patient follow-up was according to routine monitoring schedules recommended for patients receiving bedaquiline or delamanid as implemented at each endTB site. Therefore, no additional tests were performed as a part of the study. Data were to be collected in a customized, open-source electronic medical record (EMR) system developed as a part of the endTB Project across all 17 countries.

**Discussion:**

The endTB observational study will generate evidence on safety and efficacy of bedaquiline- and delamanid-containing regimens in a large, extremely heterogeneous group of MDR-TB patients, from 17 epidemiologically diverse countries. The systematic, prospective data collection of repeated effectiveness and safety measures, and analyses performed on these data, will improve the quality of evidence available to inform MDR-TB treatment and policy decisions. Further, the resources available to countries through implementation of the endTB project will have permitted countries to: gain experience with the use of these drugs in MDR-TB regimens, improve local capacity to record and report adverse events (pharmacovigilance), and enhance significantly the body of data available for safety evaluation of these drugs and other novel treatments.

**Trial registration:**

This study was registered on 24 August 2017 at clincaltrials.gov (Registration number: NCT03259269).

**Electronic supplementary material:**

The online version of this article (10.1186/s12879-019-4378-4) contains supplementary material, which is available to authorized users.

## Background

Multidrug-resistant tuberculosis (MDR-TB) remains a serious global public health problem. It is associated with high morbidity and mortality with an estimated 230,000 deaths reported globally from rifampicin resistant tuberculosis (RR-TB) or MDR-TB in 2017 alone [1]. Management of MDR-TB is complex. At the time of study development and initiation, WHO guidance recommended the use of at least 5 often expensive and highly toxic second-line tuberculosis drugs for up to 2 years [2, 3]. Moreover, only half of those treated globally have a favorable treatment outcome with these drugs [1]. The high mortality of patients with multidrug-resistant and extensively drug-resistant tuberculosis (XDR-TB) has been attributed to scarcity of safe, effective, low-cost and easy-to-deliver drugs to treat these forms of TB [4].

The MDR-TB treatment landscape is changing: For the first time in nearly half a century, two new drugs—bedaquiline and delamanid—approved by a stringent regulatory authority (SRA) for a tuberculosis indication, have offered hope for those with MDR-TB. Bedaquiline received accelerated approval by the US Food and Drug Administration in 2012; delamanid was conditionally approved by the European Medicines Agency in 2014, after results from phase IIB trials showed favorable outcomes [5, 6]. Both approvals were based on a surrogate or clinical endpoint other than survival; and Phase III trials were still pending completion at the time of approval. Following approvals from the SRA, the World Health Organization (WHO) published interim recommendations for programmatic use of bedaquiline and delamanid among eligible MDR-TB patients [7, 8]. In the meantime, country level experience with the use of bedaquiline and delamanid was also recognized as necessary to increase uptake and scale up global access. This required adoption and implementation of WHO guidance for their use in routine management of MDR-TB [9]. However, apart from some initial attempts to access these drugs through compassionate use programmes and early pilot projects for bedaquiline access, at the beginning of 2015 these drugs remained unavailable to the vast majority of patients who needed them [10].

Among the many reasons for slow uptake by countries are the cost; lack of evidence on safety of these drugs; limited experience about how best to use these drugs in combination with existing drugs; and the concern that misguided, “irrational” use of new drugs would lead to development of widespread resistance to them [11, 12]. These concerns have resulted in limited access to new drugs, as well as a dearth of efforts to optimize their use. It is estimated that only 15.7% of patients who were eligible for bedaquiline or delamanid received them during the period between 1 July 2015 and 30 June 2017 [4].

The endTB (Expand New Drug Markets for TB) Project was designed to address the implementation barriers described above and to serve as a model for more rapid integration of future treatment innovations (https://unitaid.eu/project/end-tb-project/#en). It is funded by UNITAID and implemented by Partners In Health (PIH) and its consortium partners, Médecins Sans Frontières (MSF) and Interactive Research and Development (IRD). One of the key objectives of this project was to expand programmatic access and use of new TB drugs and reduce barriers to uptake by sharing evidence to facilitate revision of national and global MDR-TB treatment guidelines. Operating in countries where consortium partners collaborate closely with National TB Programmes (NTP), endTB aimed to initiate and monitor treatment of at least 2600 MDR-TB patients with regimens containing bedaquiline or delamanid.

## Methods/design

We designed a multi-centre prospective observational cohort study to determine the safety and efficacy of bedaquiline- or delamanid-containing MDR-TB regimens used according to WHO recommendations. Patients were enrolled in 17 countries: Armenia, Bangladesh, Belarus, Democratic People’s Republic of Korea (DPRK), Ethiopia, Georgia, Haiti, Indonesia, Kazakhstan, Kenya, Kyrgyzstan, Lesotho, Myanmar, Pakistan, Peru, South Africa and Vietnam. The countries were selected to reflect the heterogeneity of high-burden MDR-TB settings globally, that is: high and low levels of second-line drug resistance, a mix of public-private health providers, high- and low-human immunodeficiency virus (HIV) prevalence, and inpatient and outpatient management of MDR-TB patients.

### Study population

All patients who started treatment with a bedaquiline- and/or delamanid-containing regimen and received clinical care at an endTB site between 1 April 2015 and 30 September 2018 were eligible.

### endTB monitoring procedures and follow-up

The endTB observational study captures routine clinical data for study participants who are treated under the supervision of the NTPs or local implementing partners at each site. No additional interventions or procedures are undertaken as part of the observational study.

**Fig. 1:**
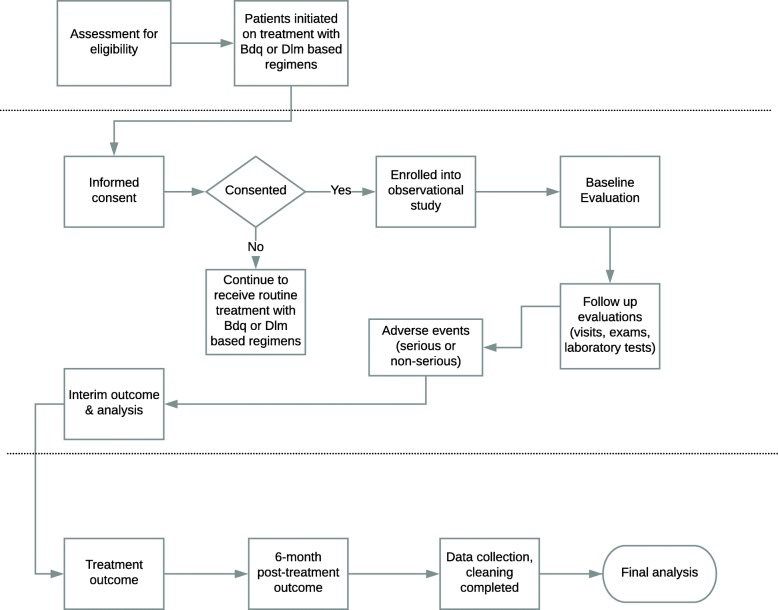
endTB observational study process-flow. Pre-enrollment stage; enrollment and follow-up; final outcome & analysis

Figure 1 illustrates the process-flow from pre-enrollment, to enrollment and follow-up, to the final outcome and analysis. The decision to start patients on a bedaquiline- or delamanid-containing regimen was made by responsible physicians according to indications in WHO interim guidance for the use of bedaquiline [7] and delamanid [8]; in some countries an expert committee or “consilium” participated in decision-making. Use of bedaquiline or delamanid was not affected in any way by participation in the observational study.

The monitoring schedule conforms to national guidelines for TB and MDR-TB treatment, reflecting guidance for regimens containing bedaquiline or delamanid. At the start of treatment, patients typically underwent a full medical history, a clinical examination, ECG, audiometry, laboratory blood tests and sputum smear microscopy, culture and drug-susceptibility testing (DST). In some countries, patients were routinely hospitalized at the beginning of treatment; in others, clinically stable patients started treatment as outpatients. After hospital discharge, patients are generally evaluated monthly, including a clinical examination, ECG, audiometry (for those receiving a second-line injectable), laboratory blood tests and sputum smear microscopy and culture. At least one post-treatment follow-up examination, along with sputum smear microscopy and culture, is planned for six months after the end of treatment. A typical monitoring schedule is shown in Additional file 1.

Treatment is observed at all study sites. Patient adherence is recorded and assessed using the treatment card routinely used by NTPs. Some sites use additional methods, such as pill counts, to track adherence. Treatment support, such as home visits, counselling, and/or social support, is provided at all sites.

### Protection of human subjects and ethical approval

This study was approved by the Partners Healthcare Human Research Committee (Boston, MA, USA), the MSF Ethics Review Board (Geneva, Switzerland), IRD Institutional Review Board (Karachi, Pakistan) and in all 17 enrolling countries by local ethics committees or IRBs. The study is registered on clinicaltrials.gov (NCT03259269).

Patients started on treatment with bedaquiline or delamanid were invited to enroll in the observational study. Informed consent was solicited from patients (or assent from the patient and consent from a guardian in the case of minors, as defined by local legal requirements). During this process, study staff clearly communicated potential risks in the appropriate language. Those who did not consent to their data being used for research still received treatment with bedaquiline or delamanid per national and site protocols. Patients were informed that they may withdraw consent at any time throughout the course of the observational study without any impact on their treatment and follow-up. Study-eligible patients whose treatment ended for any reason (e.g., treatment completion, death, loss-to-follow-up, transfer) before informed consent could be solicited may be included retrospectively. No data will be used from patients from whom consent was solicited and refused.

### Data collection and management

This study involved collection of data only, without experimental interventions or additional patient encounters with research staff outside of routine treatment visits. Local and central research staff ensured that data collection and reporting followed study protocols through site monitoring visits and regular local review of documentation. Standardized forms capture routine clinical, sociodemographic, microbiological, clinical laboratory results, and treatment records of TB and comorbidities and safety data. Local research staff performed site level quality checks of paper-based forms. Most data were directly collected into standardized forms (Additional file 2) and then entered into the endTB electronic medical record (EMR). Based on an open-source system called ‘Bahmni’ (https://www.bahmni.org), the endTB EMR (Database MySQL) was developed with an independent software company. It was designed to capture and harmonize detailed longitudinal patient-level data across all 17 countries. The endTB EMR is freely available for use by other MDR-TB programs or projects (install and set up: https://github.com/endtb/endtb-config; https://github.com/endtb/endtb-config/wiki/Deployment-Steps). Continuous quality control and quality assurance (QA) are achieved through automated range checks at data entry, real-time data reviews at each study site, periodic source-data verification, and a system of monthly data quality checks at central level. The queries generated by the central QA are sent to site staff who review, confirm entered data or make appropriate corrections to site EMRs and report to the central site for resolution of each query.

### Pharmacovigilance

As a part of endTB, a pharmacovigilance (PV) database is maintained and hosted by a single PV unit based in Geneva, Switzerland. The PV unit was established to ensure that safety information is properly reported to improve medical practices. The reporting aims to ensure the safety of patients treated with regimens containing new TB drugs and to disseminate relevant safety information to appropriate stakeholders in a timely manner. This includes:

- Reporting of Serious Adverse Events (SAE) within 24 h of physician knowledge [Serious adverse event is an untoward medical occurrence that, at any severity level: results in death; requires hospitalization or prolongation of hospitalization; results in persistent or significant disability/incapacity; is life-threatening; is a congenital anomaly or a birth defect; is otherwise medically significant]; and
- Periodic reporting of non-serious adverse events (AE) of clinical significance including: AEs of special interest, AEs recorded as having led to a treatment change/interruption, and AEs otherwise judged to be clinically significant by the treating physician.

This process is initiated when a physician sends a case report to the PV unit within 24 h of knowledge of a SAE. The physician is responsible to assess severity, outcome and causality (based on: temporal relationship, plausible pharmacological mechanism, results of suspension and re-challenge, previous reaction and lack of other plausible cause) of the SAE. After preliminary medical review at the PV unit, selected unexpected and possibly drug-related SAEs are reviewed by the medical review board (MRB) by at least 2 PV officers and 2 experienced MDR-TB experts. All SAEs are reported to the appropriate local authority, as well as to the manufacturers (for bedaquiline and delamanid), and are reviewed quarterly for evidence of unexpected safety signals. This is the first formal, multi-country post-marketing surveillance for drugs to treat MDR-TB. In addition to the monitoring of individual case safety reports, periodic analyses are performed in order to detect any change in drug safety profile and allow for timely risk minimization action implementation. Pharmacovigilance monitoring represents best practice for all post-marketing use of therapeutics. Consequently, it occurs for all patients who receive treatment in the endTB project irrespective of their participation in the observational study.

### Primary exposure

The primary exposure was the use of bedaquiline and/or delamanid. Exposure to these drugs was classified at baseline (i.e., the earliest start date of bedaquiline and/or delamanid initiation with endTB) as well as throughout the full regimen.

### Covariates

Standard variable definitions were used across all sites. Baseline variables included demographic characteristics; co-morbidities such as diabetes, cardiovascular disease, cancer, renal dysfunction, psychiatric illness, HIV, hepatitis B and C; TB risk factors, prior TB treatment and severity of TB disease; and physician-cited WHO indication that included 2 main categories for use of bedaquiline and/or delamanid: (i) patients for whom the construction of a regimen with four likely effective second-line drugs is not possible and/or (ii) patients who have high risk of unfavorable outcome [7, 8]. Repeated measures, that is those collected at both baseline and follow-up, included companion anti-TB drugs (dose, duration, changes) received during study participation; key concomitant medications; clinical (haematology, biochemistry, viral load for HIV-infected, HbA1c for diabetics) and microbiological/molecular (smear, culture, phenotypic and/or genotypic DST) laboratory testing; chest x-rays; audiometry, visual screening, and ECGs; physical exam results (including peripheral neuropathy screening). Additional details are available in the Technical Basis of the endTB Observational Study (www.endtb.org) and in the standardized forms (Additional file 2).

### Outcome variables

#### Primary outcomes

Efficacy: (i) End of treatment outcomes [13]: cured and treatment completed (favorable outcomes); (ii) treatment failed, lost to follow-up, died (unfavorable outcomes).

Safety: (i) Incidence and outcome of clinically relevant AEs. Clinically relevant AEs are: SAEs and pre-defined adverse events of special interest (QT prolongation, peripheral neuropathy, optic neuritis, myelosuppression, hearing loss, acute renal failure, hypokalemia, hypomagnesemia, hepatotoxicity, and hypothyroidism) occurring at a severity that, according to endTB clinical guidance, leads to a change in TB regimen or to supplementation (e.g., magnesium); (ii) frequency of, time to and severity grade of clinically relevant AEs.

#### Secondary outcomes

Efficacy: (i) frequency of and time to relapse; (ii) frequency of conversion during 6 months after initiation of treatment with bedaquiline or delamanid; (iii) frequency of and time to culture reversion after initial conversion; (iv) frequency of recurrence-free survival (confirmed absence of radiological, clinical or bacteriological signs of TB at the 6-month post treatment follow-up visit). Qualitative and operational aspects of endTB project implementation will also be assessed at some sites including health structure re-organisation and extra human resources required, monitoring schedule differences between sites, treatment delivery strategies and patient support (e.g. counseling, food, incentives, transport reimbursement) provided.

### Statistical considerations

#### Power/sample size

Based on annual country projections of the number of MDR- and XDR-TB patients with indications for use of the new drugs, we estimated that at least 2600 MDR-TB patients would initiate treatment with bedaquiline or delamanid at endTB sites in 17 countries. Assuming that 95% consented to participate in the observational study, this would yield a sample size of 2470 patients for analysis. Table 1 shows the 95% confidence intervals [CI] corresponding to different endpoint proportions for this sample size of 2470. For example, if there is 80% success in the study, we can conclude with 95% confidence that the true percent of success is between 78 and 82%. And, with respect to rare (toxicity) endpoints, if none are reported, we can rule out with 95% confidence a ‘true’ frequency of 0.15% or higher.

**Table 1:** Precision of endpoint estimates

Endpoint Proportion	95% CI
0	[0, 0.0015]^a^
0.05	[0.04, 0.06]
0.10	[0.09, 0.11]
0.20	[0.18, 0.22]
0.30	[0.28, 0.32]
0.40	[0.38, 0.42]
0.50	[0.48, 0.52]
0.60	[0.58, 0.62]
0.70	[0.68, 0.72]
0.80	[0.78, 0.82]
0.90	[0.89, 0.91]

^a^Exact 95 CI

### Analysis

We will calculate the frequency of primary effectiveness and safety outcomes and their respective 95% confidence intervals, using exact confidence intervals as needed. We will stratify analyses by physician-cited WHO indication for use of bedaquiline and/or delamanid [7, 8] and examine cohort effects through adjustment for calendar time and time since introduction of drugs at the study site. Secondary analyses will estimate the frequency of primary outcomes in subgroups (e.g., patients with XDR-TB) and identify patient and regimen-characteristics (e.g., use of bedaquiline and/or delamanid for greater than six months) associated with primary outcomes. Heterogeneity in local norms and prescribing practices across participating sites will facilitate comparison of different regimens and treatment strategies. Confounding, including confounding by indication, will be an important consideration in analyses of both baseline and post-baseline (i.e., on treatment) exposures. We will conduct multivariable regression analyses to adjust for factors (i.e., confounders) associated with both the primary exposure of interest (e.g., treatment strategy or regimen) and outcome. Analyses will also adjust for time-dependent confounders using inverse probability weighting, as appropriate [14]. A head-to-head comparison of bedaquiline-containing regimens and delamanid-containing regimens is not a priority since the WHO indications for use of the two drugs were different. We will account for clustering by site using robust variance estimators, generalized linear mixed models or other methods. We will use methods such as multiple imputation to account for missing covariate data.

### Study limitations

There are a number of potential limitations inherent to a prospective observational study nested in routine programmatic care for MDR-TB in 17 countries. We attempt, however, to mitigate effects of these limitations through design and/or analysis decisions. Limitations include significant expected heterogeneity of patients and practices. Heterogeneity in clinical monitoring and reporting practices was minimized by implementing a single protocol and standardized tools for data collection; standardized trainings for clinical and research staff and uniform comprehensive cross-site data quality and cleaning protocols. Moreover, selected analytic methods account for site differences. Selection bias may occur if those who refuse study participation have a different risk of unfavorable outcome than those who accept participation. To address this, we have tracked informed consent acceptance at each site and will conduct sensitivity analyses that consider various scenarios accounting for study refusal. Lastly, the intervention was not part of the study and was not randomized. Confounding by indication is likely to have occurred in the use and duration of bedaquiline and/or delamanid and companion drugs. In the design phase of this study, we collected data on the myriad of time-varying factors that could influence regimen prescription, and we will adjust for them in the analysis phase using appropriate statistical methods.

## Discussion

The overall goal of endTB is to reduce the barriers for uptake of the new TB drugs globally. One of these barriers is the lack of evidence about the efficacy and safety of bedaquiline and delamanid, which had led WHO to highly restrict the eligibility for the new TB drugs. In turn, many NTPs were reluctant to introduce them into routine use. In most countries, bedaquiline and delamanid use is restricted to the sickest patients, as a last therapeutic resort, which limits the potential benefits of these new drugs [15, 16]. The endTB observational study was specifically designed to generate critical evidence that can inform global treatment guidelines on use of bedaquiline and delamanid under programmatic conditions. The observational study focused on the programmatic implementation of treatment according to the WHO guidance in place at the time the project started. Additional, important questions about the optimization of regimens containing these—and repurposed—drugs and especially about all-oral regimens were also anticipated by the endTB project. The endTB trial (Clinicaltrials.gov identifier: NCT02754765) planned at the same time as the endTB observational study, explores these optimization questions, which are better suited to randomized studies. Although the endTB trial design was finalized prior to accumulation of safety and effectiveness results in the endTB observational study, [17, 18] ongoing evaluation of the assumptions that guided the trial sample size and revision of the control regimen composition have been informed by the endTB observational study.

While clinical trials are “the preferred source of evidence for measuring the effects of interventions” in the GRADE process for WHO Guideline development [19], observational studies of MDR-TB treatment continue to provide critical evidence that shapes our understanding of the best way to treat MDR-TB [20, 21]. Observational studies, particularly of patients treated under programmatic conditions, can have a number of potential pitfalls which we have tried to address in the design and implementation of the endTB observational study. Generating high quality evidence in programmatic conditions is challenging particularly for NTPs who are chronically underfunded for both clinical care and data reporting [22]. This is further complicated in MDR-TB treatment, which requires complex treatment regimens over a long period requiring comprehensive patient support [23]. Patients receiving these treatments often experience AEs that must be detected, managed and recorded. The endTB observational study brought additional resources for data collection, provided clinical guidance, and support for adverse event monitoring, reporting, and management. endTB observational study countries all worked in partnership with a member of the endTB consortium; most had extensive experience treating MDR-TB.

It is difficult for NTPs to fulfill the requirement to perform post-marketing pharmacovigilance for patients receiving bedaquiline and delamanid, and shortened regimens [24, 25]. In light of its critical importance to patient safety, endTB has supported PV at all of the endTB sites. In addition, the endTB observational study is designed to collect data on clinically relevant AEs due to all TB drugs, not just the new TB drugs such as bedaquiline and delamanid. This advances the overall goal of patient safety by providing systematic reporting on all clinically relevant events that occur during MDR-TB treatment. Conventional MDR-TB drugs are well known to be toxic, but, due to the weakness of national pharmacovigilance systems [26–28], very little is known about the exact frequency of AEs. Even though national guidelines may clearly stipulate the type of AE screening that should be done for all MDR-TB patients, the costs of the tests may not be covered by the NTP; if patients cannot raise funds for the tests, they are often not performed [29, 30]. In endTB, project funds were used to cover screening tests recommended by the NTP but not paid for by the health system, to ensure uniformity in screening and patient safety. In many countries this was necessary not only for new screening tests related to bedaquiline and delamanid, such as ECG, but also standard screening tests for AEs—such as hearing loss, electrolyte wasting, and peripheral neuropathy—that are commonly linked to other drugs. The endTB observational study will therefore collect data on serious and non-serious AEs, related to new and conventional MDR-TB drugs, at a scale and detail unprecedented for a programmatic setting. Exploratory analyses in endTB will examine whether recommended monitoring schedules for risks associated with bedaquiline and delamanid (e.g., QT prolongation) can be safely adjusted to facilitate integration into routine, programmatic management of MDR-TB.

The endTB observational study cohort represents one of the largest prospective, multi-country cohorts to receive programmatic treatment of MDR-TB; it is, to date, the largest, prospective multi-country cohort of patients who received programmatic treatment with bedaquiline and delamanid. It was designed to be relevant to clinicians working in ‘real life’ settings. The patient cohort is extremely heterogeneous, and includes patients from important subgroups such as XDR- and pre-XDR-TB patients; children; pregnant women; patients with extrapulmonary disease; patients with co-morbidities such as HIV, HCV, or diabetes. Such patients are commonly seen in practice, but are often ineligible for MDR-TB clinical trials. Consequently, evidence from observational research about their treatment experiences is critical. Additional value accrues from the fact that endTB patients are being treated in a variety of settings, including hospitals, health centers, and community-based care; rural and urban; private and public sectors.

Evidence generated by this study is meant to inform national and global treatment guidelines for the new TB drugs. Data will be shared periodically using various methods and fora including workshops, abstracts, presentations and publications. Through the endTB website (www.endtb.org), countries will have unrestricted access to resources developed as a part of the endTB project including e-learning modules, pharmacovigilance forms, and the *endTB Clinical and Programmatic Guide for Patient Management with New TB Drugs.* The endTB observational study and other endTB activities are expected to lower market and regulatory barriers to adoption of the new TB drugs, increase access for patients, and improve the quality of MDR-TB treatment globally.

## Additional files

Additional file 1: Typical Monitoring Schedule; schedule of patient monitoring exams and laboratory tests. (DOCX 85 kb)

Additional file 2: endTB data collection forms. (DOCX 182 kb)

## Data Availability

This is a study protocol manuscript, Not applicable.

## Abbreviations

AE: Adverse event
DST: Drug susceptibility testing
ECG: Electrocardiogram
EMA: European Medicines Association
EMR: Electronic Medical Record
endTB: Expand New Drug Markets for TB
FDA: Food and Drug Administration
GDF: Global Drug Facility
GFATM: Global Fund to Fight AIDS, TB and Malaria
HIV: Human immunodeficiency virus
IRB: Institutional Review Board
IRD: Interactive Research and Development
MDR-TB: Multidrug-resistant tuberculosis
MRB: Medical Review Board
MSF: Médecins Sans Frontières
NTP: National TB Programmes
PIH: Partners In Health
PV: Phamacovigilance
QA: Quality assurance
RR-TB: Rifampicin resistant tuberculosis
SAE: Serious adverse event
SRA: Stringent regulatory authority
TB: Tuberculosis
WHO: World Health Organization
XDR-TB: Extensively drug-resistant tuberculosis
