# Supplementary material for: The endTB observational study protocol: treatment of MDR-TB with bedaquiline or delamanid containing regimens
Source: BMC Infect Dis. 2019 Aug 20;19:733. doi: 10.1186/s12879-019-4378-4 (PMC6701145; doi:10.1186/s12879-019-4378-4)
Supplement: Supplementary file 1 — Typical Monitoring Schedule; schedule of patient monitoring exams and laboratory tests. (DOCX 85 kb) [file 12879_2019_4378_MOESM1_ESM.docx]

# Additional file 1: Typical Monitoring Schedule

|  | Baseline Visit | Week 2 | Month 1 | Month 2 | Month 3 | Month 4 | Month 5 | Month 6 | While on injectable | Until end of treatment | End of treatment | Post-treatment month 6 |
| --- | --- | --- | --- | --- | --- | --- | --- | --- | --- | --- | --- | --- |
| Vital signs | X |  | X | X | X | X | X | X | Monthly | |  |  |
| Performance status | X |  |  | X |  |  |  |  |  |  | X |  |
| Brief peripheral neuropathy screen | X |  | X | X | X | X | X | X | Monthly | | X | X |
| Audiometry | X |  | X | X | X | X | X | X | Monthly |  | X |  |
| Visual acuity and colorblindness screen | X |  | X | X | X | X | X | X | Monthly | | X | X |
| Clinical assessment | X | X | X | X | X | X | X | X | At all visits | | X | X |
| Weight | X | X | X | X | X | X | X | X | Monthly | | X |  |
| Smear | X |  | X | X | X | X | X | X | Monthly | | X | X |
| Culture | X |  | X | X | X | X | X | X | Monthly | | X | X |
| First- and second-line DST | X |  |  |  |  |  |  |  |  |  |  |  |
| DST to new TB drugs |  |  |  |  |  |  |  | If culture-positive | | | | |
| Electrocardiogram | X | X | X | X | X | X | X | X |  |  | X | X |
| Full blood count, AST, ALT | X | X | X | X | X | X | X | X | Monthly | | X |  |
| Urea, creatinine, serum potassium | X |  | X | X | X | X | X | X | Monthly |  | X |  |
| TSH | X |  |  |  | X |  |  |  | Every 3 months | |  |  |
| HBsAg, HCVAb, HbA1c | X |  |  |  |  |  |  |  |  |  |  |  |
| Pregnancy test | X |  |  |  |  |  |  |  |  |  |  |  |
| HIV serostatus, CD4, Viral load | X |  |  |  |  |  |  |  |  |  |  |  |
| Chest X-Ray | X |  |  |  |  |  |  | X |  |  | X |  |
