# Supplementary material for: The endTB observational study protocol: treatment of MDR-TB with bedaquiline or delamanid containing regimens
Source: BMC Infect Dis. 2019 Aug 20;19:733. doi: 10.1186/s12879-019-4378-4 (PMC6701145; doi:10.1186/s12879-019-4378-4)
Supplement: Supplementary file 2 — endTB data collection forms. (DOCX 182 kb) [file 12879_2019_4378_MOESM2_ESM.docx]

**Additional file 2: endTB forms**

**Baseline Assessment**

**REGISTRATION AND DEMOGRAPHICS**

| Surname: ____________________ Given name: ______________________ | |
| --- | --- |
| EMR ID#:  __ __ __ — __ __ __ — __ __ __ __ __ | |
| Date of birth: __ __ /__ __ __ /__ __ __ __ (e.g. DD/MMM/YYYY) | |
| Age: __ __ years | Gender: ☐ Male   ☐ Female |

**CONTACT DETAILS**

| Address: _________________________  Permanent residence district: _________________________  Permanent residence country: ________________________  Telephone #: _________________________ |
| --- |

**TREATMENT REGISTRATION**

| TB Register: ☐ Basic Management Unit TB register ☐ Second line TB register | |
| --- | --- |
| Date of registration in DRTB National program: __ __ /__ __ __ /__ __ __ __ | |
| Registration Number: _______________ | Registration Facility: _______________ |

**SOCIAL HISTORY**

| Date of baseline assessment: __ __ /__ __ __ /__ __ __ __ (e.g. DD/MMM/YYYY) |
| --- |

| Marital status (mark one): | ☐ Married  ☐ Living together  ☐ Single  ☐ Divorced  ☐ Widowed  ☐ Separated  ☐ Other:_____________ |
| --- | --- |
| Homeless within past year? | ☐ Yes   ☐ No   ☐ Unknown |
| Current employment status (mark one): | ☐ Employed  ☐ Unable to work  ☐ Student  ☐ Unemployed  ☐ Housework  ☐ Pensioner  ☐ Other:________________ |
| Is the patient a refugee, displaced or migrant? | ☐ Yes   ☐ No   ☐ Unknown |
| Has the patient ever been in prison? | ☐ Yes   ☐ No   ☐ Unknown |
| If YES: When was the patient in prison? | ☐ Currently   ☐ In the past |
| Has the patient ever been a health worker? | ☐ Never   ☐ In the past  ☐ Currently   ☐ Unknown |

| Does the patient drink alcohol? | ☐ Yes   ☐ No  ☐ Unknown |
| --- | --- |
| If YES: How many standard alcoholic drinks does the patient drink per week? _______ | |
| Does the patient smoke at least 1 cigarette per day? | ☐ Yes   ☐ No  ☐ Unknown |
| Has the patient used intravenous drugs in the past year? | ☐ Yes   ☐ No  ☐ Unknown |
| Has the patient used non-prescribed, non-injectable drugs in the past year? (e.g. cannabis, cocaine, prescription stimulants without a prescription, methamphetamine, inhalants, sedatives, hallucinogens, street opioids) | ☐ Yes   ☐ No  ☐ Unknown |

**FOOD SECURITY (optional)**

| In the past 30 days, was there ever no food to eat of any kind in your house because of lack of resources to get food? | ☐ Never  ☐ Rarely (1-2 times)  ☐ Sometimes (3-10 times)  ☐ Often (more than 10 times) |
| --- | --- |
| In the past 30 days, did you or any household member go to sleep at night hungry because there was not enough food? | ☐ Never  ☐ Rarely (1-2 times)  ☐ Sometimes (3-10 times)  ☐ Often (more than 10 times) |
| In the past 30 days, did you or any household member go a whole day and night without eating anything at all because there was not enough food? | ☐ Never  ☐ Rarely (1-2 times)  ☐ Sometimes (3-10 times)  ☐ Often (more than 10 times) |

**TB HISTORY**

| Has the patient ever been treated for TB in the  past? | ☐ Yes   ☐ No  ☐ Unknown |
| --- | --- |
| If YES, What was the year of the start of patient's first TB treatment? | __ __ __ __  (e.g. 2015) or  ☐ Unknown  ☐ Unknown |
| Has the patient ever had more than 1 month of treatment for **drug-susceptible** TB in the past? | ☐ Yes   ☐ No |
| If YES,  How many times did the patient start **drug-susceptible** TB treatment?  ☐ 1   ☐ 2  ☐ 3   ☐ 4    ☐ 5 or more  What is the registration number of the most recent **drug-susceptible** TB treatment episode?  _____________________ or   ☐ Unknown  What is the outcome of the most recent **drug-susceptible** TB treatment?  ☐ Cured  ☐ Completed  ☐ Failed  ☐ Lost to follow-up  ☐ Not evaluated (unknown or transferred out)  ☐ Treatment adapted (moved to DR-TB register)  In which registration facility was the patient registered for the most recent **drug-susceptible** TB treatment? ____________ | |
| Has the patient ever had more than 1 month of treatment for **drug-resistant** TB in the past? | ☐ Yes   ☐ No  ☐ Unknown |
| If YES,  How many times did the patient start **drug-resistant** TB treatment?  ☐ 1   ☐ 2   ☐ 3   ☐ 4   ☐ 5 or more  What is the registration number of the most recent **drug-resistant** TB treatment episode?  _____________________ or   ☐ Unknown  What is the outcome of the most recent **drug-resistant** TB treatment?  ☐ Cured  ☐ Completed  ☐ Failed  ☐ Lost to follow-up  ☐ Not evaluated (unknown or transferred out or transferred back to DS TB)  ☐ Treatment adapted (empirical treatment that was ended due to DST results  showing resistance to second line drugs and therefore rendering the current  treatment sub-optimal or ineffective)  In which registration facility was the patient registered for the most recent **drug-resistant** TB treatment? ____________ | |

**Drugs taken for greater than one month (circle all that apply)**

| **Group 1** | isoniazid | **Group 4** | cycloserine |
| --- | --- | --- | --- |
|  | rifampicin |  | ethionamide |
|  | rifabutin |  | para-aminosalicylic acid |
|  | rifapentine |  | para-aminosalicylate sodium |
|  | ethambutol |  | prothionamide |
|  | pyrazinamide |  | terizidone |
| **Group 2** | amikacin | **Group 5** | amoxicillin/clavulanate |
|  | capreomycin |  | bedaquiline |
|  | kanamycin |  | clarithromycin |
|  | streptomycin |  | clofazimine |
| **Group 3** | ciprofloxacin |  | delamanid |
|  | gatifloxacin |  | imipenem/cilastatin |
|  | levofloxacin |  | linezolid |
|  | moxifloxacin |  | meropenem |
|  | ofloxacin |  | thioacetazone |
| Other, specify: __________________   Other, specify: ____________________ | | | |
| Other, specify: __________________   Other, specify: ____________________ | | | |

| **Previous TB treatment episodes (optional):** | | | | |
| --- | --- | --- | --- | --- |
| **Start date** | **End date** | **Type** | **Regimen (abbreviation)** | **Outcome** |
|  |  |  |  |  |
|  |  |  |  |  |
|  |  |  |  |  |
|  |  |  |  |  |

**PAST MEDICAL HISTORY (CO-MORBIDITIES)**

| Any known drug allergies | ☐ Yes   ☐ No   ☐ Unknown  If yes, please specify: ________________ |
| --- | --- |
| Confirmed HIV serostatus (For HIV diagnosis, CD4 count, ARV initiation; if date is not known exactly then use the first day of the month, if month unknown then use July) | ☐ Positive   ☐ Negative   ☐ Unknown  If POSITIVE:  Diagnosis date: __ __ /__ __ __ /__ __ __ __  Last CD4 count: _________ cells/mm^3^  ☐ Unknown  Date of CD4: __ __ /__ __ __ /__ __ __ __  Last RNA viral load: _______copies/ml  ☐ Unknown  Date of viral load: __ __ /__ __ __ /__ __ __ __  Date of ARV initiation: __ __ /__ __ __ /__ __ __ __  Currently taking ARV treatment:  ☐ Yes  ☐ No ☐ Unknown  Current ARV regimen:  ______ /______ /______ / ______ |
| Diabetes (type I or II) | ☐ Yes   ☐ No   ☐ Unknown  If YES, last HbA1c: ______ |
| Chronic renal disease | ☐ Yes   ☐ No   ☐ Unknown |
| Hepatic cirrhosis | ☐ Yes   ☐ No   ☐ Unknown |
| Chronic obstructive pulmonary disease | ☐ Yes   ☐ No   ☐ Unknown |
| Cancer | ☐ Yes   ☐ No   ☐ Unknown  If YES, type: _____________________________ |
| Heart disease or atherosclerotic disease (e.g. heart failure, heart attack, stroke) | ☐ Yes   ☐ No   ☐ Unknown  If YES, type: _____________________________ |
| Confirmed Hepatitis B | ☐ Yes   ☐ No   ☐ Unknown |
| Confirmed Hepatitis C | ☐ Yes   ☐ No   ☐ Unknown |
| Depression | ☐ Yes   ☐ No   ☐ Unknown |
| Other psychiatric illness | ☐ Yes   ☐ No   ☐ Unknown  If YES, type: _____________________________ |
| Seizure disorder (chronic) | ☐ Yes   ☐ No   ☐ Unknown |
| Existing Neuropathy (optional)  If YES, neuropathy grading: | ☐ Yes ☐ No ☐ Unknown  ☐ 1 ☐ 2 ☐ 3 ☐ 4 |
| Other pre-existing diseases: (including TB surgeries) |  |

**PHYSICAL EXAMINATION**

| Date of physical examination | __ __ /__ __ __ /__ __ __ __ (DD/MM/YYYY) |
| --- | --- |
| Weight (kg): __ __ __.__ | Height (cm): __ __ __ |
| Pulse (beats per minute): __ __ __ | Respiratory rate (per minute): __ __ __ |

**Brief peripheral neuropathy screen**

| Normal | Mild  ----------------------------------------------------------------------------------------------  Severe | | | | | | | | | |
| --- | --- | --- | --- | --- | --- | --- | --- | --- | --- | --- |
| 00 | 01 | 02 | 03 | 04 | 05 | 06 | 07 | 08 | 09 | 10 |

| **1. Subjective symptoms (write score for right and left legs)** | **Left** | **Right** |
| --- | --- | --- |
| a. Pain, aching, or burning in feet, legs |  |  |
| b. "Pins and needles" in feet, legs |  |  |
| c. Numbness (lack of feeling) in feet, legs |  |  |

**2. Vibration perception (write score for right and left legs)**


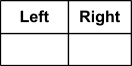

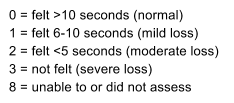


**3. Ankle reflexes (write score for right and left legs)**


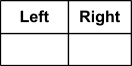

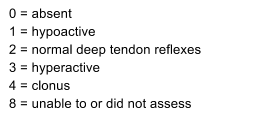


**Visual acuity**

| Left eye | 20 / __ __ __ |
| --- | --- |
| Right eye | 20 / __ __ __ |

**Colorblindness screen (Ishihara test)**

Write the number of correct plates from 1-11 in the book of 14 plates.

|  | Number |
| --- | --- |
| Left eye |  |
| Right eye |  |

**OR**

**Simplified Colorblindness Screen**

| Ishihara result: | ☐ Normal       ☐ Abnormal |
| --- | --- |

**CASE DEFINITION**

| WHO registration group | ☐ New  ☐ Relapse  ☐ Treatment after loss to follow-up  ☐ Treatment after failure  ☐ Other previously treated patients |
| --- | --- |
| History of past anti-TB drug use  (if registration group is not NEW) | ☐ Previously treated only with first line drugs  ☐ Previously treated with second line drugs  ☐ History unclear/unknown |
| Disease site | ☐ Pulmonary  ☐ Extrapulmonary, exact site:     ____________________________ |
| Detection of *M. tuberculosis*? | ☐ Bacteriologically confirmed  ☐ Not confirmed, clinically diagnosed |
| If diagnosis was "bacteriologically confirmed", what was the method of confirmation (mark all that apply)? | ☐ Sputum smear positive  ☐ Xpert MTB/RIF positive for *M. tuberculosis*  ☐ Hain test positive for *M. tuberculosis*  ☐ Culture (solid or MGIT) positive for *M. tuberculosis*  ☐ Other test: ____________________ |
| Drug resistance | ☐ Profile: unconfirmed  ☐ Confirmed drug susceptible  ☐ Confirmed drug resistant TB  ☐ Unknown |
| Subclassification of drug resistance profile (mark only one) | ☐ H (S) resistance  ☐ HE(S) resistance  ☐ R resistance with H susceptibility  ☐ Xpert MTB/RIF rifampicin resistance only  ☐ Confirmed MDR  ☐ Confirmed pre-XDR (FQ)  ☐ Confirmed pre-XDR (Inj)  ☐ Confirmed XDR  ☐ Other |
| MDR-TB or rifampicin resistance diagnosis date  (date of first result indicating MDR or rifampicin resistance; could be micro/molecular test or clinical diagnosis of MDR-TB from endTB or non-endTB site): | __ __ /__ __ __ /__ __ __ __ |

| Sputum tests ordered at this assessment: | ☐ Smear  ☐ Culture  ☐ DST |
| --- | --- |

| Next assessment date: __ __ /__ __ __ /__ __ __ __ (DD/MMM/YYYY)  Reason for next assessment (check one):  ☐ 2 week assessment  ☐ Planned monthly assessment visit: Month ___ ___  ☐ Other assessment: Reason ________________________ |
| --- |
| Form filled by: _______________________  Date: __ __ /__ __ __ /__ __ __ __ |
| Form entered by: ____________________   Date: __ __ /__ __ __ /__ __ __ __ |

**Treatment Initiation**

| Surname: _____________________ Given name: ______________________  Facility patient ID#: _________________  EMR ID#: __ __ __ — __ __ __ — __ __ __ __ __  Registration number: ___________________________ |
| --- |

**NEW DRUGS TREATMENT ELIGIBILITY**

| Is this patient eligible for treatment with new drugs (according to WHO indication)? | ☐ Yes ☐ No ☐ Unknown |
| --- | --- |
| If YES, date that patient was determined to be eligible for new drugs: | __ __ /__ __ __ /__ __ __ __ (DD/MMM/YYYY) |

**If YES, what is the indication for new TB drugs (check all that apply)?**

| Patients for whom the construction of a regimen with four likely effective second-line drugs is not possible (check all that apply): | ☐ XDR (resistance to a fluoroquinolone and at least one second-line injectable)  ☐ Pre-XDR - fluoroquinolone (resistance to a fluoroquinolone, but susceptible to second-line injectables)  ☐ Pre-XDR - injectable (resistance to at least one second-line injectable, but susceptible to a fluoroquinolone)  ☐ Other patterns of resistance that are not XDR or pre-XDR (two or more Group 4 drugs)  ☐ Contact with a patient infected with a strain with one of the above resistance patterns  ☐ Unable to tolerate MDR drugs necessary for construction of the regimen  ☐ Previously "failed" an MDR regimen |
| --- | --- |
| Other patients who have high risk of unfavorable outcome but who do not fit the above categories (check all that apply): | ☐ Extensive or advanced disease  ☐ Co-morbidities or other conditions such as drug contraindications, patients with low body mass index (BMI), HIV, diabetes  ☐ Patients from catchment areas that have poor MDR-TB treatment outcomes despite good programmatic conditions (e.g. sites with extensive second-line drug resistance background) |

**What is the situation of the patient (optional)?**

| Mark the situation which best applies: | ☐ **Situation 1:** The patient is starting a new regimen that contains new drugs. In this situation, when the patient starts the new regimen, the previous treatment should be closed and the appropriate outcome should be recorded.  ☐ **Situation 2:** If an empiric regimen for a duration longer than a month is being changed due to the results of a baseline DST (i.e. the treatment is "adapted" to the baseline DST results), the previous treatment should be closed and the outcome should be recorded as "Treatment adapted".  ☐ **Situation 3:** If the regimen is being changed due to adverse events (e.g. replacement of kanamycin with bedaquiline) but the patient is culture negative and does not fulfill the definition of failure, this is not considered a new treatment, but rather a continuation of the original treatment. |
| --- | --- |
| Additional comments (about why the patient is being started on new TB drugs): | |

**CONSENT**

| Has the Treatment with New Drugs Consent Form been explained and signed? | ☐ Yes  ☐ No  ☐ Unknown |
| --- | --- |
| Has the endTB Observational Study Consent Form been explained and signed? | ☐ Yes, signed  ☐ No, refused  ☐ Pending to be asked  ☐ Consent not possible (patient died, lost to follow-up, treatment completed, or transferred out) |

**Pregnancy and Breastfeeding Status (at treatment initiation)**

| Is the patient or partner pregnant at the time of starting treatment? | ☐ Yes ☐ No ☐ Not applicable  ☐ Unknown |
| --- | --- |
| If YES, what is the estimated date of delivery? __ __ /__ __ __ /__ __ __ __ | |
| Is the patient or partner breastfeeding at the time of starting treatment? | ☐ Yes ☐ No ☐ Not applicable  ☐ Unknown |

**TREATMENT START**

| Did the patient start treatment? | ☐ Yes (fill out Active Medication Log) ☐ No |
| --- | --- |
| **Treatment start date:** | __ __ /__ __ __ /__ __ __ __ (e.g. DD/MMM/YYYY) |
| Is this treatment start date estimated? | If so, please check the appropriate box(es):  ☐ Day estimated  ☐ Month estimated  ☐ Year estimated |
| If YES, in which facility did they start their treatment?  (This is the facility where the patient is registered, not necessarily the facility where the patient receives treatment.) | Facility name: _____________________________  Facility patient ID # ________________________  Facility district: ____________________________  Facility city: ______________________________  Facility country: ___________________________ |
| If NO, reason for not starting treatment: | ☐ Patient refused  ☐ Lost to follow-up  ☐ Died  (date of death __ __ /__ __ __ /__ __ __ __)  ☐ Rejected by committee  ☐ Referred to other facility  ☐ Other |

**Next Assessment**

| Date of next assessment: | __ __ /__ __ __ /__ __ __ __ |
| --- | --- |
| Reason for next assessment: | ☐ 2 week assessment  ☐ Planned monthly assessment  ☐ Other assessment |
| Reason for other assessment | ________________ |

| Form filled by: _______________________ Date: __ __ /__ __ __ /__ __ __ __ |
| --- |
| Form entered by: ____________________ Date: __ __ /__ __ __ /__ __ __ __ |

**Active Medication Log**

| Surname: ______________ Given name: ________________ | Facility patient ID#: _________________ | EMR ID#: __ __ __ — __ __ __ — __ __ __ __ __ |
| --- | --- | --- |

***Instructions:*** *Record all medication changes (e.g. medication stopped or has a change in dose) on a new line.*

| **Drug abbreviations:**  Isoniazid (H)  Rifampicin (R)  Ethambutol (E)  Pyrazinamide (Z)  Streptomycin (S)  Amikacin (Am) | Kanamycin (Km)  Capreomycin (Cm)  Levofloxacin (Lfx)  Moxifloxacin (Mfx)  Prothionamide (Pto)  Ethionamide (Eto)  Cycloserine (Cs) | Terizidone (Trd)  Para-aminosalicylic acid (PAS)  Bedaquiline (Bdq)  Delamanid (Dlm)  Linezolid (Lzd)  Clofazimine (Cfz)  Imipenem/Cilastatin (Imp/Cln)  Amoxicillin/clavulanate (Amx/Clv)  Other _______________ | ***Reason for medication change (Anti-TB drugs):**  1. planned change (e.g. dose change, end of injectable, end of treatment)  2. adverse event (e.g.changes due to contraindications)  3. reintroduction/replacement of stopped drug  4. resistance to drug  5. drug supply or drug administration issue  6. pregnancy  7. other:____________  ****Reason for drug administration (non-TB drugs or concomitant medication):**  1. Adverse event  2. Comorbidity  3. Severe condition of patient  4. Other medical or treatment related ____________ | **AE ID#:** Write AE ID # (see AE form) if change is related to an AE, otherwise write "NA" |
| --- | --- | --- | --- | --- |

**Anti-TB drugs**

|  | **Anti-TB drug** | **Total daily dose (mg)** | **Route**  **(Oral (PO), Intramuscular (IM), Intravenous (IV), Inhalation)** | **Schedule**  **(6 days per week, 7 days per week, every other day, 3 days per week, other)** | **Additional Instructions** | **Start date**  DD / MMM / YYYY | **Stop date**  DD / MMM / YYYY | **Reason for medication change*** | **AE ID#** |
| --- | --- | --- | --- | --- | --- | --- | --- | --- | --- |
| 1 |  |  |  |  |  |  |  |  |  |
| 2 |  |  |  |  |  |  |  |  |  |
| 3 |  |  |  |  |  |  |  |  |  |
| 4 |  |  |  |  |  |  |  |  |  |
| 5 |  |  |  |  |  |  |  |  |  |
| 6 |  |  |  |  |  |  |  |  |  |
| 7 |  |  |  |  |  |  |  |  |  |
| 8 |  |  |  |  |  |  |  |  |  |
| 9 |  |  |  |  |  |  |  |  |  |
| 10 |  |  |  |  |  |  |  |  |  |
| 11 |  |  |  |  |  |  |  |  |  |
| 12 |  |  |  |  |  |  |  |  |  |
| 13 |  |  |  |  |  |  |  |  |  |
| 14 |  |  |  |  |  |  |  |  |  |
| 15 |  |  |  |  |  |  |  |  |  |
| 16 |  |  |  |  |  |  |  |  |  |
| 17 |  |  |  |  |  |  |  |  |  |
| 18 |  |  |  |  |  |  |  |  |  |
| 19 |  |  |  |  |  |  |  |  |  |
| 20 |  |  |  |  |  |  |  |  |  |
| 21 |  |  |  |  |  |  |  |  |  |
| 22 |  |  |  |  |  |  |  |  |  |
| 23 |  |  |  |  |  |  |  |  |  |
| 24 |  |  |  |  |  |  |  |  |  |
| 25 |  |  |  |  |  |  |  |  |  |
| 26 |  |  |  |  |  |  |  |  |  |
| 27 |  |  |  |  |  |  |  |  |  |
| 28 |  |  |  |  |  |  |  |  |  |
| 29 |  |  |  |  |  |  |  |  |  |

**Concomitant medication log**

|  | **Drug Name** | **Drug Quantity (units)** | **Formulation** | **Route** | **Frequency** | **Start date**  DD / MMM / YYYY | **Stop date**  DD / MMM / YYYY | **Reason for drug administration**** | **AE ID#** |
| --- | --- | --- | --- | --- | --- | --- | --- | --- | --- |
| 1 |  |  |  |  |  |  |  |  |  |
| 2 |  |  |  |  |  |  |  |  |  |
| 3 |  |  |  |  |  |  |  |  |  |
| 4 |  |  |  |  |  |  |  |  |  |
| 5 |  |  |  |  |  |  |  |  |  |
| 6 |  |  |  |  |  |  |  |  |  |
| 7 |  |  |  |  |  |  |  |  |  |
| 8 |  |  |  |  |  |  |  |  |  |
| 9 |  |  |  |  |  |  |  |  |  |
| 10 |  |  |  |  |  |  |  |  |  |
| 11 |  |  |  |  |  |  |  |  |  |
| 12 |  |  |  |  |  |  |  |  |  |
| 13 |  |  |  |  |  |  |  |  |  |
| 14 |  |  |  |  |  |  |  |  |  |
| 15 |  |  |  |  |  |  |  |  |  |
| 16 |  |  |  |  |  |  |  |  |  |
| 17 |  |  |  |  |  |  |  |  |  |
| 18 |  |  |  |  |  |  |  |  |  |
| 19 |  |  |  |  |  |  |  |  |  |
| 20 |  |  |  |  |  |  |  |  |  |
| 21 |  |  |  |  |  |  |  |  |  |
| 22 |  |  |  |  |  |  |  |  |  |
| 23 |  |  |  |  |  |  |  |  |  |
| 24 |  |  |  |  |  |  |  |  |  |
| 25 |  |  |  |  |  |  |  |  |  |
| 26 |  |  |  |  |  |  |  |  |  |
| 27 |  |  |  |  |  |  |  |  |  |
| 28 |  |  |  |  |  |  |  |  |  |
| 29 |  |  |  |  |  |  |  |  |  |

**Follow-up Assessment**

| Surname: _____________________ Given name: ______________________  Facility patient ID#: _________________  EMR ID#:  __ __ __ — __ __ __ — __ __ __ __ __ |
| --- |

| Assessment date: __ __ /__ __ __ /__ __ __ __ (DD/MMM/YYYY) | |
| --- | --- |
| Treatment facility name: __________________________________ | |
| Type of assessment (check one only) | ☐ 2 week assessment  ☐ Planned monthly assessment: Month___ __  ☐ Other assessment  ☐ End of treatment assessment  ☐ 6 Month post-treatment assessment |

**CLINICAL EXAMINATION**

| Weight (kg): __ __ __.__ | Height (cm): __ __ __ |
| --- | --- |
| Pulse (beats per minute): __ __ __ | Respiratory rate (per minute): __ __ __ |

| Is the patient (if female) or the patient's (if male) partner currently pregnant? | ☐ Yes   ☐ No   ☐ Unknown  ☐ NA |
| --- | --- |
| If YES, write the PV Pregnancy form Case ID# | _____________________ |

**Brief peripheral neuropathy screen**

| Normal | Mild  ----------------------------------------------------------------------------------------------------  Severe | | | | | | | | | |
| --- | --- | --- | --- | --- | --- | --- | --- | --- | --- | --- |
| 00 | 01 | 02 | 03 | 04 | 05 | 06 | 07 | 08 | 09 | 10 |

| **1. Subjective symptoms (write score for right and left legs)** | **Left** | **Right** |
| --- | --- | --- |
| a. Pain, aching, or burning in feet, legs |  |  |
| b. "Pins and needles" in feet, legs |  |  |
| c. Numbness (lack of feeling) in feet, legs |  |  |

**2. Vibration perception (write score for right and left legs)**


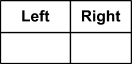

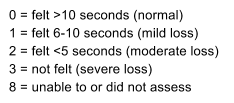


**3. Ankle reflexes (write score for right and left legs)**


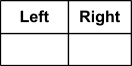

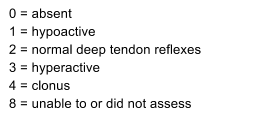


**Visual acuity**

| Left eye | 20 /__ __ __ |
| --- | --- |
| Right eye | 20 /__ __ __ |

**Colorblindness screen (Ishihara test)**

Write the number of correct plates from 1-11 in the book of 14 plates.

|  | Number |
| --- | --- |
| Left eye |  |
| Right eye |  |

**OR**

**Simplified Colorblindness screen**

| Ishihara screen result: | ☐ Normal       ☐ Abnormal |
| --- | --- |

**ADVERSE EVENTS ASSESSMENT AND TB REGIMEN CHANGES**

| Are you reporting a new AE?  If YES, write AE ID # | ☐ Yes   ☐ No  _______________ |
| --- | --- |
| If reporting a new AE, is it an SAE?  If YES, write SAE ID: | ☐ Yes   ☐ No  _______________ |
| Does the patient have an ongoing AE/SAE that does not have a final outcome (check AE log)? | ☐ Yes   ☐ No |
| Has there been a change in the TB regimen or concomitant medications, including dosage adjustment, stopping a medication, or adding a new medication? | ☐ Yes   ☐ No |

| Sputum tests ordered at this assessment: | ☐ Smear  ☐ Culture  ☐ DST  ☐ Unknown |
| --- | --- |

| Does the patient drink alcohol? | ☐ Yes   ☐ No   ☐ Unknown |
| --- | --- |
| If YES: How many standard alcoholic drinks does the patient drink per week? _______ | |

**CENTRAL VENOUS ACCESS DEVICE (CVAD) (Optional)**

| **Date of CVAD Insertion:** | | __ __ /__ __ __ /__ __ __ __ |
| --- | --- | --- |
| **Type of CVAD:** | ☐ PACC   ☐ PICC   ☐ OTHER | |
| **Drugs by CVAD:** | ☐ Imp   ☐ Imp+Amx/Clv   ☐ Imp+Amx/Clv+Am ☐ Imp+Amx/Clv+Cm   ☐ Non Currently   ☐ Other | |
| **Date of CVAD Removal:** | | __ __ /__ __ __ /__ __ __ __ |
| **Reason for CVAD Removal:** | | ☐ No longer needed   ☐ Complication    ☐ Patient Request ☐ Other |

| Next assessment date: __ __ /__ __ __ /__ __ __ __ (DD/MM/YYYY)  Reason for next assessment (check one):  ☐ 2 week assessment  ☐ Planned monthly assessment visit: Month ___ ___  ☐ Other assessment: Reason ________________________  ☐ End of treatment assessment  ☐ 6 Month post-treatment assessment |
| --- |

| Form filled by: _______________________  Date: __ __ /__ __ __ /__ __ __ __ |
| --- |
| Form entered by: ____________________   Date: __ __ /__ __ __ /__ __ __ __ |

**Outcome**

*Instructions: This form is filled out whenever there is an outcome: after a patient dies; when a patient is judged to be lost to follow-up; when it is decided to stop treatment because of treatment failure; or on the last day of treatment in the case of cure or completed.*

| Surname: _____________________ Given name: ______________________  Facility patient ID#: _________________  EMR ID#: __ __ __ — __ __ __ — __ __ __ __ __ | |
| --- | --- |
| Date of the end of treatment (the last day the patient received treatment): | __ __ /__ __ __ /__ __ __ __ |
| Date of end of treatment decision (the day outcome was declared): | __ __ /__ __ __ /__ __ __ __ |

*Tick only one of the outcomes in the left column, then answer the corresponding questions in the right column.*

| **Outcome**  **(tick one)** | **Definitions and additional questions** |
| --- | --- |
| ☐ Cured | *Treatment completed as recommended by the national policy without evidence of failure AND 3 three or more consecutive cultures taken at least 30 days apart are negative after the intensive phase.* |
| ☐ Completed | *Treatment completed as recommended by the national policy without evidence of failure BUT no record that three or more consecutive cultures taken at least 30 days apart are negative after the intensive phase.* |
| ☐ Died | Date of death: __ __ /__ __ __ /__ __ __ __  Suspected primary cause of death (check only one option):  ☐ TB is immediate cause of death  ☐ TB is contributing cause of death  ☐ Surgery-related death (type of surgery: __________________)  ☐ Cause other than TB (suspected cause: _________________)  ☐ Cause related to TB treatment  ☐ Unknown |
| ☐ Failed | *Treatment terminated or need for permanent regimen change of at least two anti-TB drugs.*  Reason for treatment failure (check all that apply):  ☐ Lack of conversion  ☐ Bacteriological reversion after conversion to negative  ☐ Evidence of additional acquired resistance to fluoroquinolones or second-line injectable drugs  ☐ Adverse drug reactions  ☐ Other: ____________________________ |
| ☐ Lost to follow-up | *Treatment was interrupted for two consecutive months or more.*  Why was the patient's treatment interrupted (check all that apply)?  ☐ Patient refused to finish treatment/Bad relation with health worker  ☐ Substance abuse  ☐ Social problem: family, financial, complex social situation  ☐ Left region, country  ☐ Adverse events  ☐ No confidence in treatment  ☐ Unknown  ☐ Other:______________________________  Comments_________________________________________ |
| ☐ Not evaluated | *No treatment outcome is assigned (this includes cases transferred out to another treatment unit and whose treatment outcome is unknown).*  Did the patient transfer out? ☐ Yes ☐ No  If YES, to where? ______________________________________  If NO, why does the patient have this outcome?  ___________________________________________________ |

| Form filled by: _______________________ Date: __ __ /__ __ __ /__ __ __ __ |
| --- |
| Form entered by: ____________________ Date: __ __ /__ __ __ /__ __ __ __ |

**Post-treatment Month 6 Outcome**

*Instructions: This form is filled out at the 6 month post-treatment visit once test results are available. It can be filled out earlier if a post treatment outcome occurs before this visit (as in the case of a patient who was cured but then dies 3 months after finishing treatment).*

| Surname: _____________________ Given name: ______________________  Facility patient ID#: _________________  EMR ID#: __ __ __ — __ __ __ — __ __ __ __ __ | |
| --- | --- |
| Date of post-treatment outcome decision: | __ __ /__ __ __ /__ __ __ __ |

*Tick only one of the outcomes in the left column, then answer the corresponding questions in the right column.*

| **Outcome**  **(tick one)** | **Definitions and additional questions** |
| --- | --- |
| ☐ No change in outcome post-treatment | *Patient was:*   - *cured or completed and is now culture-negative with no signs of relapse; or* - *failed treatment, and has not died or been to lost to follow-up since.* |
| ☐ Died post-treatment | *Patient died after finishing treatment.*  Date of death: __ __ /__ __ __ /__ __ __ __  Suspected primary cause of death (check only one option):  ☐ TB is immediate cause of death  ☐ TB is contributing cause of death  ☐ Surgery-related death (type of surgery: __________________)  ☐ Cause other than TB (suspected cause: _________________)  ☐ Cause related to TB treatment  ☐ Unknown |
| ☐ Relapse or recurrence | *Patient was given a treatment outcome of "cured" or "completed" at the end of treatment, and now has TB diagnosed again by a clinician.* |
| ☐ Lost to follow-up post-treatment | *Post-treatment follow-up was not possible.*  Why was the patient lost to follow-up (check all that apply)?  ☐ Patient refused follow-up/Bad relation with health worker  ☐ Substance abuse  ☐ Social problem: family, financial, complex social situation  ☐ Left region, country  ☐ Adverse events  ☐ No confidence in treatment  ☐ Unknown  ☐ Other:______________________________  Comments_________________________________________ |
| ☐ Not evaluated | *No post-treatment outcome is assigned (this includes cases transferred out to another treatment unit and whose post-treatment outcome is unknown).*  Did the patient transfer to another facility for post-treatment follow-up? ☐ Yes ☐ No  If YES, to where? ______________________________________  If NO, why does the patient have this outcome?  ___________________________________________________ |

| Form filled by: _______________________ Date: __ __ /__ __ __ /__ __ __ __ |
| --- |
| Form entered by: ____________________ Date: __ __ /__ __ __ /__ __ __ __ |

**Adverse Event Form AE ID #: ________**

| Surname: _____________________ Given name: ______________________  Facility patient ID#: _________________  EMR ID#: __ __ __ — __ __ __ — __ __ __ __ __ | |
| --- | --- |
| Date of onset of event: __ __ /__ __ __ /__ __ __ __ (DD/MMM/YYYY) | |
| Date of reporting the event (today's date): __ __ /__ __ __ /__ __ __ __ | |
| Were all anti-TB drugs suspended due to this AE? | ☐ Yes ☐ No |

#### Use one AE form per event. Tick the box in the right column that applies to the AE being reported.

| **Organ system** | **Common Adverse Events**  **(check ONE)** |
| --- | --- |
| *Cardiovascular disorders* | ☐ Cardiac rhythm  ☐ **Prolonged (corrected) QT interval** |
| *Chemistry* | ☐ **Hypokalemia (K ≤ 3.4 mEq/L)**  ☐ Hypomagnesemia (Mg ≤ 1.4 mmol/L)  ☐ Lactate (serum lactate greater than ULN) |
| *Ear disorders* | ☐ **Hearing impairment (hearing loss)**  ☐ Tinnitus  ☐ Vestibular disorder |
| *Endocrine disorders* | ☐ **Hypothyroidism** |
| *Enzymes* | ☐ **Increased liver enzymes (ALT increased or AST increased (≥ 1.1 x ULN))** |
| *Eye disorders* | ☐ **Optic nerve disorder (optic neuritis)** |
| *Gastrointestinal disorders* | ☐ Diarrhea  ☐ Dyspepsia  ☐ Nausea  ☐ Oral discomfort/dysphagia  ☐ Pancreatitis  ☐ Vomiting |
| *Hematology* | ☐ **Absolute neutrophil count low (ANC ≤ 1500/mm^3^)**  ☐ **Anemia (Hb < 10.5 g/dL)**  ☐ **Platelets decreased (< 75,000/mm^3^)** |
| *Immune disorders* | ☐ Allergic reaction |
| *Musculoskeletal disorders* | ☐ Arthralgia  ☐ Arthritis  ☐ Myalgia  ☐ Tendinopathy |
| *Neurological disorders* | ☐ Dysgeusia  ☐ Headache  ☐ Peripheral neuropathy (neurosensory disorder or paresthesia)  ☐ Seizure |
| *Reproductive system and breast disorders* | ☐ Gynecomastia |
| *Psychiatric disorders* | ☐ Anxiety  ☐ Depression  ☐ Psychosis  ☐ Suicidal ideation |
| *Renal and urinary disorders* | ☐ **Acute kidney injury (acute renal failure)** |
| *Skin disorders* | ☐ Mucocutaneous symptoms (includes rash)  ☐ Pruritus  ☐ Skin hypo- or hyper-pigmentation |
| ***Other adverse events*** | |
| *Other (enter one adverse event) if not listed in the most common list:* | _________________________________ |

**Severity**

| Grade | ☐ 1 ☐ 2 ☐ 3 ☐ 4 |
| --- | --- |

**Related test results**

| **Test** | **Lab ID number** | **Date** | **Value** |
| --- | --- | --- | --- |
|  |  | __ __ /__ __ __ /__ __ __ __ |  |
|  |  | __ __ /__ __ __ /__ __ __ __ |  |
|  |  | __ __ /__ __ __ /__ __ __ __ |  |

| Form filled by: _______________________ Date: __ __ /__ __ __ /__ __ __ __ |
| --- |
| Form entered by: ____________________ Date: __ __ /__ __ __ /__ __ __ __ |

**Adverse Event Outcome AE ID #: ________**

| Surname: _____________________ Given name: ______________________  Facility patient ID#: _________________  EMR ID#: __ __ __ — __ __ __ — __ __ __ __ __ |
| --- |

| Did this AE become an SAE?  If yes, SAE Case ID # | ☐ Yes ☐ No  __________________ |
| --- | --- |
| Date of AE outcome | __ __ /__ __ __ /__ __ __ __ (DD/MMM/YYYY) |
| Outcome of this AE | ☐ Fatal (fill out an SAE form)  ☐ Not resolved  ☐ Resolved  ☐ Resolved with sequelae  ☐ Resolving  ☐ Unknown |

**Severity**

| Maximum severity grade | ☐ 1 ☐ 2 ☐ 3 ☐ 4 |
| --- | --- |

**Causal Factors: Anti-TB drugs**

| Is this adverse event related to any of the TB drugs in the patient’s regimen? | ☐ Yes ☐ No |
| --- | --- |

| **Anti-TB drugs** | **Possibly Related to AE?** | **Final action taken** |
| --- | --- | --- |
| Drug 1: ____________________ | ☐ Yes  ☐ No | ☐ Dose maintained (no changes)  ☐ Dose reduced  ☐ Drug permanently withdrawn  ☐ Unknown |
| Drug 2: ____________________ | ☐ Yes  ☐ No | ☐ Dose maintained (no changes)  ☐ Dose reduced  ☐ Drug permanently withdrawn  ☐ Unknown |
| Drug 3: ____________________ | ☐ Yes  ☐ No | ☐ Dose maintained (no changes)  ☐ Dose reduced  ☐ Drug permanently withdrawn  ☐ Unknown |
| Drug 4: ____________________ | ☐ Yes  ☐ No | ☐ Dose maintained (no changes)  ☐ Dose reduced  ☐ Drug permanently withdrawn  ☐ Unknown |
| Drug 5: ____________________ | ☐ Yes  ☐ No | ☐ Dose maintained (no changes)  ☐ Dose reduced  ☐ Drug permanently withdrawn  ☐ Unknown |

**Other Causal Factors**

| Are there any other causal factors, such co-morbidities, procedures, other non-TB drugs, etc.? (check ALL that apply) | ☐ Non TB drugs  ☐ Comorbidity  ☐ Other |
| --- | --- |

| **Related other non-TB drugs (ART, diabetes drugs, etc.)** |
| --- |
| 1. |
| 2. |
| 3. |

| **Related co-morbidities (chronic kidney problem, diabetes, etc.)** |
| --- |
| 1. |
| 2. |
| 3. |

| **Other related or causal factors (procedure, etc.)** |
| --- |
| 1. |
| 2. |

| Form filled by: _______________________ Date: __ __ /__ __ __ /__ __ __ __ |
| --- |
| Form entered by: ____________________ Date: __ __ /__ __ __ /__ __ __ __ |

**Hospital Admission**

| Surname: _____________________ Given name: ______________________  Facility patient ID#: _________________  EMR ID#: __ __ __ — __ __ __— __ __ __ __ __ |
| --- |
| Date of hospital admission: __ __ /__ __ __ /__ __ __ __ (DD/MMM/YYYY) |
| Hospital name: ________________________________________ |
| Admission diagnosis (optional):_______________________________________ |

| Other notes or comments: |
| --- |

| Form filled by: _______________________ Date: __ __ /__ __ __ /__ __ __ __ |
| --- |
| Form entered by: ____________________ Date: __ __ /__ __ __ /__ __ __ __ |

**Hospital Discharge Summary**

| Surname: _____________________ Given name: ______________________  Facility patient ID#: _________________  EMR ID#: __ __ __ — __ __ __— __ __ __ __ __ | |
| --- | --- |
| Hospital name: ________________________________________ | |
| Date of admission: __ __ /__ __ __ /__ __ __ __ (DD/MMM/YYYY) | |
| Date of discharge: __ __ /__ __ __ /__ __ __ __ (DD/MMM/YYYY) | |
| Main reason for hospitalization (mark only one) | ☐ Treatment initiation  ☐ Infection control  ☐ Adverse event  ☐ Co-morbidity  ☐ Severe clinical condition  ☐ Surgical operation  ☐ Patient behavior  ☐ Social reason  ☐ Other: _______________________________  ☐ Unknown |
| Discharge diagnosis (final reason for hospital admission):  ________________________________________________________________ | |

| Are you reporting a NEW AE?  If YES, enter the AE ID number here: | ☐ Yes ☐ No  ___________ |
| --- | --- |
| If reporting a NEW AE, is this a NEW SAE?  If YES, enter the SAE ID number here: | ☐ Yes ☐ No  ___________ |
| Does the patient have an ongoing adverse event that does not have a final outcome? | ☐ Yes ☐ No |
| Has there been a change in the TB regimen or concomitant medications, including dosage adjustment, stopping a medication, or adding a new medication? | ☐ Yes ☐ No |

| Hospital course (optional): |
| --- |
| Other notes or comments: |

**TB-related surgery**

| Did the patient receive TB-related surgery during the hospital admission? | ☐ Yes ☐ No  If YES, date of the TB-related surgery:  __ __ /__ __ __ /__ __ __ __ |
| --- | --- |
| Mark the type of surgery: | ☐ Pneumonectomy  ☐ Lobectomy  ☐ Segmental or wedge resection  ☐ Decortication  ☐ Thoracostomy  ☐ Other:______________________ |
| Indication for surgery: | ☐ Failure of culture conversion  ☐ Destroyed or trapped lung  ☐ Complicated/persistent cavit(ies)  ☐ Massive hemoptysis  ☐ Empyema or pleural effusion  ☐ Re-operation for complication  ☐ Other:______________________ |

| Form filled by: _______________________ Date: __ __ /__ __ __ /__ __ __ __ |
| --- |
| Form entered by: ____________________ Date: __ __ /__ __ __ /__ __ __ __ |

**Bacteriology Results**

| Surname: _____________________ Given name: ______________________  Facility patient ID#: _________________  EMR ID#: __ __ __ — __ __ __— __ __ __ __ __ |
| --- |

| Type of assessment | ☐ Baseline assessment  ☐ Planned monthly assessment visit: Month ___ ___  ☐ Other assessment  ☐ End of treatment assessment  ☐ 6 Month post-treatment assessment |
| --- | --- |

**SAMPLE COLLECTION**

| Sample collection date: __ __ /__ __ __ /__ __ __ __ (DD/MMM/YYYY)  Laboratory name:_______________________________  Sample ID#: ______________________________ | |
| --- | --- |
| Sample type (check one): | ☐ Sputum  ☐ Lymph node  ☐ Cerebrospinal fluid (CSF)  ☐ Gastric aspirate  ☐ Urine  ☐ Pleural fluid  ☐ Ascites fluid  ☐ Bone biopsy  ☐ Other tissue biopsy  ☐ Bronchial lavage  ☐ Unknown |

**SMEAR MICROSCOPY**

| Date smear done: | __ __ /__ __ __ /__ __ __ __ or ☐ Not done |
| --- | --- |
| Smear test lab ID #: | ______________________________ |
| Result:: | ☐ Negative  ☐ Scanty 1-3  ☐ Scanty 4-9  ☐ 1+  ☐ 2+  ☐ 3+ or more |

**XPERT MTB/RIF**

| Date Xpert MTB/RIF test done: | __ __ /__ __ __ /__ __ __ __ or ☐ Not done |
| --- | --- |
| Xpert MTB/RIF test lab ID #: | ______________________________ |
| *M. tuberculosis* complex: | ☐ Detected  ☐ Not detected  ☐ Invalid  ☐ No result  ☐ Error |
| If *M. tuberculosis* DETECTED, then burden is: | ☐ Very low  ☐ Low  ☐ Medium  ☐ High |
| Rifampicin resistance: | ☐ Detected  ☐ Not detected  ☐ Indeterminate |

**HAIN MTBDRplus**

| Date HAIN MTBDRplus test done: | __ __ /__ __ __ /__ __ __ __ or ☐ Not done |
| --- | --- |
| HAIN MTBDRplus test lab ID #: | __________________________ |
| *M. tuberculosis* complex: | ☐ Detected  ☐ Not detected |
| If *M. tuberculosis* complex DETECTED, complete the following: | |
| HAIN MTBDRplus isoniazid: | ☐ Susceptible  ☐ Resistant  ☐ Indeterminate |
| HAIN MTBDRplus rifampicin: | ☐ Susceptible  ☐ Resistant  ☐ Indeterminate |

**HAIN MTBDRsl**

| Date HAIN MTBDRsl test done: | __ __ /__ __ __ /__ __ __ __ or ☐ Not done |
| --- | --- |
| HAIN MTBDRsl test ID #: | ___________________________ |
| *M. tuberculosis* complex: | ☐ Detected  ☐ Not detected |
| If *M. tuberculosis* complex DETECTED, complete the following: | |
| MTBDRsI fluoroquinolone: | ☐ Susceptible  ☐ Resistant  ☐ Indeterminate |
| MTBDRsI injectable: | ☐ Susceptible  ☐ Resistant  ☐ Indeterminate |

**CULTURE**

| Date culture was inoculated: | __ __ /__ __ __ /__ __ __ __ or ☐ Not done |
| --- | --- |
| Culture test lab ID #: | __________________________ |
| Type of media: | ☐ Lowenstein-Jensen  ☐ MGIT  ☐ Middlebrook 7H11S  ☐ TLA  ☐ Other: ______________ |
| Culture results: | ☐ Positive for *M.tuberculosis* complex  ☐ Negative for *M. tuberculosis* complex  ☐ Contaminated  ☐ Only positive for non-tuberculous mycobacteria  ☐ Other |
| If solid media culture is positive for *M. tuberculosis*, mark the colony count: | ☐ <10 colonies  ☐ 1+ (10-100)  ☐ 2+ (>100)  ☐ 3+ (>200)  ☐ Not done |

**DRUG SUSCEPTIBILITY**

| Type of media: | ☐ Lowenstein-Jensen  ☐ MGIT 960/320  ☐ Middlebrook 7H11S  ☐ TLA  ☐ Other: _____________ |
| --- | --- |

| Drug | Result (mark one) | | |
| --- | --- | --- | --- |
|  | Susceptible | Resistant | Indeterminate |
| Isoniazid (0.2 µg/ml) |  |  |  |
| Isoniazid (1.0 µg/ml) |  |  |  |
| Rifampicin |  |  |  |
| Ethambutol |  |  |  |
| Pyrazinamide |  |  |  |
| Streptomycin |  |  |  |
| Kanamycin |  |  |  |
| Capreomycin |  |  |  |
| Ofloxacin |  |  |  |
| Levofloxacin |  |  |  |
| Moxifloxacin (0.5 µg/ml) |  |  |  |
| Moxifloxacin (2.0 µg/ml) |  |  |  |
| Amikacin |  |  |  |
| Ethionamide |  |  |  |
| Cycloserine |  |  |  |
| PAS |  |  |  |
| Other: __________________ |  |  |  |
| Other: __________________ |  |  |  |
| Other: __________________ |  |  |  |
| Other: __________________ |  |  |  |
| Other: __________________ |  |  |  |
| Other: __________________ |  |  |  |

| Form filled by: _______________________ Date: __ __ /__ __ __ /__ __ __ __ |
| --- |
| Form entered by: ____________________ Date: __ __ /__ __ __ /__ __ __ __ |

**Laboratory Results**

| Surname: _____________________ Given name: ______________________  Facility patient ID#: _________________  EMR ID#: __ __ __ — __ __ __— __ __ __ __ __ |
| --- |

| Type of assessment | ☐ Baseline assessment  ☐ 2 week assessment  ☐ Planned monthly assessment: Month ___ ___  ☐ Other assessment  ☐ End of treatment assessment  ☐ 6 Month post-treatment assessment |
| --- | --- |

**Hematology**

| **Sample Details**  Sample collection date: __ __ /__ __ __ /__ __ __ __ (DD/MMM/YYYY)  Lab name: ___________________________  Sample ID#: ___________________________ | | | |
| --- | --- | --- | --- |
|  | ***Result*** | ***Unit*** | ***Abnormal?*** |
| Hemoglobin |  | ☐ mmol/L  ☐ g/dL | ☐ Yes  ☐ No |
| Hematocrit |  | % | ☐ Yes  ☐ No |
| Platelet count |  | x10^9^/L | ☐ Yes  ☐ No |
| RBC count |  | x10^12^/L | ☐ Yes  ☐ No |
| WBC count |  | x10^9^/L | ☐ Yes  ☐ No |
| Neutrophils |  | % | ☐ Yes  ☐ No |
| Absolute neutrophil count (ANC) |  | x10^9^/L | ☐ Yes  ☐ No |

**Chemistry**

| **Sample Details**  Sample collection date: __ __ /__ __ __ /__ __ __ __ (DD/MMM/YYYY)  Lab name: ___________________________  Sample ID#: ___________________________ | | | |
| --- | --- | --- | --- |
|  | ***Result*** | ***Unit*** | ***Abnormal?*** |
| Potassium |  | ☐ mg/dL  ☐ mmol/L | ☐ Yes  ☐ No |
| Magnesium |  |  | ☐ Yes  ☐ No |
| Ionised calcium |  |  | ☐ Yes  ☐ No |
| Urea |  |  | ☐ Yes  ☐ No |
| Creatinine |  |  | ☐ Yes  ☐ No |
| Glucose (fasting) |  | ☐ mg/dL  ☐ mmol/L | ☐ Yes  ☐ No |
| Glucose (non fasting) |  | ☐ mg/dL  ☐ mmol/L | ☐ Yes  ☐ No |
| HbA1c |  |  | ☐ Yes  ☐ No |
| TSH |  | mIU/L | ☐ Yes  ☐ No |
| Amylase |  |  | ☐ Yes  ☐ No |
| Lipase |  |  | ☐ Yes  ☐ No |
| Serum albumin |  | ☐ g/L  ☐ μmol/L | ☐ Yes  ☐ No |

|  | ***Result*** | ***Upper limit of normal*** | ***Unit*** | ***Abnormal?*** |
| --- | --- | --- | --- | --- |
| AST/SGOT |  |  | IU/L | ☐ Yes  ☐ No |
| ALT/SGPT |  |  | IU/L | ☐ Yes  ☐ No |
| Total bilirubin |  |  | ☐ μmol/L  ☐ mg/dL | ☐ Yes  ☐ No |

**Serological and other tests**

| **Sample Details**  Sample collection date: __ __ /__ __ __ /__ __ __ __ (DD/MMM/YYYY)  Lab name: ___________________________  Sample ID#: ___________________________ | | |
| --- | --- | --- |
|  | ***Result*** | ***Unit*** |
| Pregnancy test | ☐ Positive  ☐ Negative  ☐ Indeterminate |  |
| HIV test | ☐ Positive  ☐ Negative  ☐ Indeterminate |  |
| CD4 count |  | ☐ cells/mm^3^ ☐ x10^9^/L |
| RNA viral load |  | copies/ml |
| Hep B surface antigen | ☐ Reactive ☐ Non-reactive  ☐ Borderline ☐ Pending |  |
| Hep C antibody | ☐ Reactive ☐ Non-reactive  ☐ Borderline ☐ Pending |  |

**Other tests**

| **Sample Details**  Sample collection date: __ __ /__ __ __ /__ __ __ __ (DD/MMM/YYYY)  Lab name: ___________________________  Sample ID#: ___________________________ | | | |
| --- | --- | --- | --- |
| ***Test name*** | ***Result*** | ***Unit*** | ***Abnormal?*** |
| Other test 1:__________ |  |  | ☐ Yes  ☐ No |
| Other test 2:__________ |  |  | ☐ Yes  ☐ No |
| Other test 3:__________ |  |  | ☐ Yes  ☐ No |

| Form filled by: _______________________ Date: __ __ /__ __ __ /__ __ __ __ |
| --- |
| Form entered by: ____________________ Date: __ __ /__ __ __ /__ __ __ __ |

**Chest X-ray & Radiology**

| Surname: _____________________ Given name: ______________________  EMR ID#: LSO — __ __ __— __ __ __ __ __ |
| --- |
| Date of assessment: __ __ /__ __ __ /__ __ __ __ (DD/MMM/YYYY) |

**Chest X-Ray**

| Chest X-ray ID#: ___________________________ | |
| --- | --- |
| Type of assessment | ☐ Baseline assessment  ☐ Other assessment  ☐ End of treatment assessment |

**Results (Mark one option for each question below)**

| Extent of disease: | ☐ Normal  ☐ Unilateral disease  ☐ Bilateral disease  ☐ Abnormal - extent not defined |
| --- | --- |
| Cavity size (aggregate): | ☐ No cavities  ☐ < 5 cm  ☐ ≥ 5 cm |
| Presence of fibrosis: | ☐ None  ☐ In 1 lobe or less  ☐ In more than 1 lobe |
| Comparison with last X-ray: | ☐ Improved  ☐ Worsening  ☐ Same (unchanged)  ☐ Not applicable |

**Other tests (ultrasound, CT, MRI etc.)**

| Test Name | Results |
| --- | --- |
| 1. __________________ |  |
| 2.___________________ |  |
| 3.___________________ |  |

| Form filled by: _______________________ Date: __ __ /__ __ __ /__ __ __ __ |
| --- |
| Form entered by: ____________________ Date: __ __ /__ __ __ /__ __ __ __ |

Audiometry

| Surname: _____________________ Given name: ______________________  Facility patient ID#: _________________  EMR ID#: __ __ __ — __ __ __— __ __ __ __ __ |
| --- |

| Date of audiometry: __ __ /__ __ __ /__ __ __ __ (DD/MMM/YYYY) | |
| --- | --- |
| Type of assessment: | ☐ Baseline assessment  ☐ Planned monthly assessment visit: Month ___ ___  ☐ Other assessment  ☐ End of treatment assessment  ☐ 6 Month post-treatment assessment |

| (Optional) Type of audiometry:  (e.g. Hearscreen, ShoeBox, name of private audiologist) |
| --- |

**Threshold Search Audiometry**

| **Frequency (Hz)** | **Left ear (dB)** | **Right ear (dB)** |
| --- | --- | --- |
| 250 Hz |  |  |
| 500 Hz |  |  |
| 1000Hz |  |  |
| 2000Hz |  |  |
| 4000Hz |  |  |
| 6000Hz |  |  |
| 8000Hz |  |  |

*Classification of hearing loss (based on Pure Tone Average - the average threshold values at all frequencies):*

- *Normal: 0 to 20 db*
- *Mild: 21 to 40 dB*
- *Moderate: 41 to 55 dB*
- *Moderately severe: 56 to 70 dB*
- *Severe: 71 to 90 dB*
- *Profound: 91+ dB*

***OR***

**Screening Audiometry (optional)**

| Left Ear | ☐ Normal ☐ Abnormal ☐ Unknown |
| --- | --- |
| Right Ear | ☐ Normal ☐ Abnormal ☐ Unknown |

**Adverse Event Reporting**

| Are you reporting abnormal audiometry as an adverse event? | ☐ Yes ☐ No ☐ Unknown |
| --- | --- |
| If YES, what is the AE ID number? | ___________________ |

| Form filled by: _______________________ Date: __ __ /__ __ __ /__ __ __ __ |
| --- |
| Form entered by: ____________________ Date: __ __ /__ __ __ /__ __ __ __ |

**Electrocardiogram**

| Surname: _____________________ Given name: ______________________  Facility patient ID#: _________________  EMR ID#: __ __ __ — __ __ __— __ __ __ __ __ |
| --- |

| Date of ECG: __ __ /__ __ __ /__ __ __ __ (DD/MMM/YYYY) | |
| --- | --- |
| Type of assessment | ☐ Baseline assessment  ☐ 2 week assessment  ☐ Planned monthly assessment visit: Month ___ ___  ☐ Other assessment  ☐ End of treatment assessment  ☐ 6 Month post-treatment assessment |

| Rhythm (check one) | ☐ Sinus rhythm  ☐ Atrial fibrillation  ☐ Premature ventricular complexes  ☐ Other _____________________ |
| --- | --- |

| Heart rate | ____________ beats per minute (bpm) |
| --- | --- |
| QT interval | _____________ milliseconds (ms) |
| QT_c_F interval  [*Note: QT_c_F = QT / ^3^√RR]* | _____________ milliseconds (ms) |

**Adverse Event Reporting (if ECG related AE)**

| Are you reporting an abnormal ECG as an Adverse Event? | ☐ Yes ☐ No ☐ Unknown |
| --- | --- |
| If yes, what is the AE ID #? | ____________________ |

| Form filled by: _______________________ Date: __ __ /__ __ __ /__ __ __ __ |
| --- |
| Form entered by: ____________________ Date: __ __ /__ __ __ /__ __ __ __ |

**Monthly Treatment Completeness**

| Surname: _____________________ Given name: ______________________  Facility patient ID#: _________________  EMR ID#: __ __ __ — __ __ __— __ __ __ __ __ |
| --- |

| Treatment period | Month: |  | Year: |  |
| --- | --- | --- | --- | --- |

| Treatment delivery method (most applicable this month) | ☐ DOT inpatient  ☐ DOT outpatient facility-based  ☐ DOT outpatient community-based  ☐ Self Administered Treatment (SAT)  ☐ Combination SAT and DOT  ☐ Other: _______________________________ |
| --- | --- |

| Ideal total treatment days in the month | Non-prescribed days | Prescribed days | | Principal reason for not having 100% completeness |
| --- | --- | --- | --- | --- |
|  |  | Missed | Incomplete |  |
|  |  |  |  |  |

*Main reasons for treatment for less than 100% completeness (select only one):

1. Program related (e.g. drug shortage, staff absent, other program related)
2. Medical or treatment related (e.g. adverse event, comorbidity, severe condition of patient, other medical or treatment related)
3. Patient related (e.g. family problem, social problem, financial problem, related to work, substance abuse, other patient related)
4. Other (give reasons below)

**Additional details about principal reason for less than 100% completeness:**

|  |
| --- |

**Additional contributing reasons for less than 100% completeness:**

|  |
| --- |

**DOT rate per drug (optional)**

| Anti-TB drug | **Prescribed** (*f*) (days or doses) | **Missed prescribed** (*g*) (days or doses) | **Observed** (*h*) (days or doses) | **DOT rate observed/ prescribed %**  *i = h/f x 100 or*  *i = (f-g)/f x 100* |
| --- | --- | --- | --- | --- |
|  |  |  |  |  |
|  |  |  |  |  |
|  |  |  |  |  |
|  |  |  |  |  |
|  |  |  |  |  |

| Form filled by: _______________________ Date: __ __ /__ __ __ /__ __ __ __ |
| --- |
| Form entered by: ____________________ Date: __ __ /__ __ __ /__ __ __ __ |

**Performance Status**

| Surname: _____________________ Given name: ______________________  Facility patient ID#: _________________  EMR ID#: __ __ __ — __ __ __ — __ __ __ __ __ |
| --- |

| Date of assessment: __ __ /__ __ __ /__ __ __ __ (DD/MMM/YYYY) | |
| --- | --- |
| Type of assessment | ☐ Baseline assessment  ☐ Planned monthly assessment  ☐ Other assessment  ☐ End of treatment assessment  ☐ 6 month post-treatment assessment |

**ECOG Performance Status (circle one)**

| 0 | Fully active, able to carry on all pre-disease performance without restriction |
| --- | --- |
| 1 | Restricted in physically strenuous activity but ambulatory and able to carry out work of a light or sedentary nature, i.e., light housework, office work |
| 2 | Ambulatory and capable of all self-care but unable to carry out any work activities. Up and about more than 50% of waking hours |
| 3 | Capable of only limited self-care, confined to bed or chair more than 50% of waking hours |
| 4 | Completely disabled. Cannot carry out any self-care. Totally confined to bed or chair |

| Form filled by: _______________________ Date: __ __ /__ __ __ /__ __ __ __ |
| --- |
| Form entered by: ____________________ Date: __ __ /__ __ __ /__ __ __ __ |

| **Patient information (mother)** | | | | |
| --- | --- | --- | --- | --- |
| **Patient n°:** | **Mother initials:** | **Mother date of birth:**  ____ / _____ / ________ (*dd/Mmm/yyyy*) | **Mother height:**  …………... cm | **Mother weight:**  …….……. kg |
| **[ Father** ☐ **Mother** ☐ **]** |  |  |  |  |

| **Relevant drug(s) exposure before/during pregnancy** | | | | | | | |
| --- | --- | --- | --- | --- | --- | --- | --- |
| Drug name (INN) | ………………………………… | ……………………………. | …………………………… | …………………………… | ……………………………… | ……………………………… | ……………………………… |
| Daily dose & route |  |  |  |  |  |  |  |
| Batch number |  |  |  |  |  |  |  |
| Treatment start date (*dd/Mmm/yyyy*) | ____ / _____ / ________ | ____ / _____ / ________ | ____ / _____ / ________ | ____ / _____ / ________ | ____ / _____ / ________ | ____ / _____ / ________ | ____ / _____ / ________ |
| Treatment stop date (*dd/Mmm/yyyy*) | ____ / _____ / ________ | ____ / _____ / ________ | ____ / _____ / ________ | ____ / _____ / ________ | ____ / _____ / ________ | ____ / _____ / ________ | ____ / _____ / ________ |
| Drug taken by | Father ☐ / Mother ☐ | Father ☐ / Mother ☐ | Father ☐ / Mother ☐ | Father ☐ / Mother ☐ | Father ☐ / Mother ☐ | Father ☐ / Mother ☐ | Father ☐ / Mother ☐ |
| Action taken in response to the pregnancy | | | | | | | |
| Dosage maintained | ☐ | ☐ | ☐ | ☐ | ☐ | ☐ | ☐ |
| Dose reduced | ☐ | ☐ | ☐ | ☐ | ☐ | ☐ | ☐ |
| New daily dose |  |  |  |  |  |  |  |
| On (*dd/Mmm/yyyy*) | ____ / _____ / ________ | ____ / _____ / ________ | ____ / _____ / ________ | ____ / _____ / ________ | ____ / _____ / ________ | ____ / _____ / ________ | ____ / _____ / ________ |
| Drug permanently withdrawnOn (*dd/Mmm/yyyy*) | ☐ | ☐ | ☐ | ☐ | ☐ | ☐ | ☐ |
|  | ____ / _____ / ________ | ____ / _____ / ________ | ____ / _____ / ________ | ____ / _____ / ________ | ____ / _____ / ________ | ____ / _____ / ________ | ____ / _____ / ________ |
| Drug interrupted | ☐ | ☐ | ☐ | ☐ | ☐ | ☐ | ☐ |
| From (*dd/Mmm/yyyy*) | ____ / _____ / ________ | ____ / _____ / ________ | ____ / _____ / ________ | ____ / _____ / ________ | ____ / _____ / ________ | ____ / _____ / ________ | ____ / _____ / ________ |
| To (*dd/Mmm/yyyy*) | ____ / _____ / ________ | ____ / _____ / ________ | ____ / _____ / ________ | ____ / _____ / ________ | ____ / _____ / ________ | ____ / _____ / ________ | ____ / _____ / ________ |
| Not applicable | ☐ | ☐ | ☐ | ☐ | ☐ | ☐ | ☐ |

| Pregnancy information | | | |
| --- | --- | --- | --- |
| Date of 1^st^ day of last menstrual period | ____ / _____ / ________ (*dd/Mmm/yyyy*) | Estimated date of delivery | ____ / _____ / ________(*dd/Mmm/yyyy*) |
| Pregnancy test | ☐ Positive urine test **Date**: **____ / _____ / ________** (*dd/Mmm/yyyy*) | ☐ **Positive blood test**  **Date**: **____ / _____ / ________** (*dd/Mmm/yyyy*) | ☐ **Positive ultrasound** Date: ____ / _____ / ________ (*dd/Mmm/yyyy*) |
| Pregnancy outcome | | | |
| 1. **Did the patient experience any complication during pregnancy?** | ☐ **Yes. Specify**: | | |
|  | ☐ **No** | | |
| 1. **Did the patient give birth to (a) live infant(s)?** | ☐ **Yes. Date of delivery** (*dd/Mmm/yyyy*): **____ / _____ / ________** | | |
|  | ☐ **No. Specify reason:** | | |
| 1. **Was the infant normal at birth?** | **☐ Yes** | | |
|  | **☐ No. Specify abnormality and reason:** | | |
| **Additional comment on pregnancy/delivery** |  | | |

| **Infant(s) information** | | | | | | |
| --- | --- | --- | --- | --- | --- | --- |
| **Infant number** | **Infant sex** | **Infant length** *(cm)* | **Infant weight** *(g)* | **APGAR score** | **Exposure during breastfeeding** | **Comment** |
| **1** | **F ☐ M ☐** |  |  |  | **Yes ☐ No ☐** |  |
| **2** | **F ☐ M ☐** |  |  |  | **Yes ☐ No ☐** |  |
| **3** | **F ☐ M ☐** |  |  |  | **Yes ☐ No ☐** |  |

| **Relevant medical history** *(with focus on relevant prior gynaecological/obstetric history)* |  |
| --- | --- |

| **Reporter** | | | | |
| --- | --- | --- | --- | --- |
| **Name of reporter:** | **Role in trial/program:** | **Date of awareness:**  **____ / _____ / ________** | **Address:**  **Email:**  **Phone:** | **Date and signature***:*  **____ / _____ / ________** |

**Case number:**

| **SERIOUS ADVERSE EVENT (SAE) REPORT FORM** | | |
| --- | --- | --- |
| **Sponsor:** Médecins Sans Frontières | **Protocol/Program n°:** | **Site n° (for studies) or country:** |
| **Initial report:** □ | **Follow-up report:** □ | **Date of report:** ____ / _____ / ________ (dd/Mmm/yyyy) |

| **Patient information** | | | | | |
| --- | --- | --- | --- | --- | --- |
| **Patient n°:** | **Initials:** | **Date of birth:** ____ / _____ / ________ (*dd/Mmm/yyyy*) | **Gender:** F ☐ M ☐ | **Height:** …………... cm | **Weight:** …………... kg |

| Serious adverse event(s) information | | SAE 1 | SAE 2 | | | SAE 3 |
| --- | --- | --- | --- | --- | --- | --- |
| Adverse event term | | ………………..………………………….......... | ………………..………………………….......... | | | ………………..………………………….......... |
| Event onset date (*dd/Mmm/yyyy*) | | ____ / _____ / ________ | ____ / _____ / ________ | | | ____ / _____ / ________ |
| Date event became serious (*dd/Mmm/yyyy*) | | ____ / _____ / ________ | ____ / _____ / ________ | | | ____ / _____ / ________ |
| Event end date (*dd/Mmm/yyyy*) | | ____ / _____ / ________ | ____ / _____ / ________ | | | ____ / _____ / ________ |
| Duration if <1 day (*hrs/min*) | | ____ / _____ | ____ / _____ | | | ____ / _____ |
| **Seriousness criteria** | **Death** | ☐ | ☐ | | | ☐ |
|  |  | *In case of death:* | | Death date: ____ / _____ / ________ | Autopsy: Yes ☐ No ☐ | |
|  | Life-threatening | ☐ | ☐ | | | ☐ |
|  | Hospitalization required / prolonged | ☐ | ☐ | | | ☐ |
|  |  | *Hospitalization dates:* | | Admission: ____ / _____ / ________ | Discharge: ____ / _____ / ________ | |
|  | Persistent or significant disability / incapacity | ☐ | ☐ | | | ☐ |
|  | Congenital anomaly / birth defect | ☐ | ☐ | | | ☐ |
|  | Otherwise medically important | ☐ | ☐ | | | ☐ |
| Non-serious reportable information | | ☐ | ☐ | | | ☐ |
| Severity | | Grade 1 ☐ 2☐ 3☐ 4☐ | Grade 1 ☐ 2☐ 3☐ 4☐ | | | Grade 1 ☐ 2☐ 3☐ 4☐ |
| Event outcome | Fatal | ☐ | ☐ | | | ☐ |
|  | Not resolved | ☐ | ☐ | | | ☐ |
|  | Resolved | ☐ | ☐ | | | ☐ |
|  | Resolved with sequelae | ☐ | ☐ | | | ☐ |
|  | Resolving | ☐ | ☐ | | | ☐ |
|  | Unknown | ☐ | ☐ | | | ☐ |

| **Suspected drug(s)** | **Drug 1** | **Drug 2** | **Drug 3** | **Drug 4** | **Drug 5** | **Drug 6** | **Drug 7** |
| --- | --- | --- | --- | --- | --- | --- | --- |
| **Suspected drug name (INN)** | …………………………….... | …………………………….... | …………………………….... | …………………………….... | …………………………….... | …………………………….... | …………………………….... |
| **Daily dose & route** |  |  |  |  |  |  |  |
| **Batch number** |  |  |  |  |  |  |  |
| **Treatment start date** (*dd/Mmm/yyyy*) | ____ / _____ / ________ | ____ / _____ / ________ | ____ / _____ / ________ | ____ / _____ / ________ | ____ / _____ / ________ | ____ / _____ / ________ | ____ / _____ / ________ |
| **Treatment stop date** (*dd/Mmm/yyyy*) | ____ / _____ / ________ | ____ / _____ / ________ | ____ / _____ / ________ | ____ / _____ / ________ | ____ / _____ / ________ | ____ / _____ / ________ | ____ / _____ / ________ |
| **Action taken in response to the event** | | | | | | | |
| **Dose maintained** | **☐** | **☐** | **☐** | **☐** | **☐** | **☐** | **☐** |
| **Dose reduced** | **☐** | **☐** | **☐** | **☐** | **☐** | **☐** | **☐** |
| **New daily dose** |  |  |  |  |  |  |  |
| **On** (*dd/Mmm/yyyy*) | ____ / _____ / ________ | ____ / _____ / ________ | ____ / _____ / ________ | ____ / _____ / ________ | ____ / _____ / ________ | ____ / _____ / ________ | ____ / _____ / ________ |
| **Drug permanently withdrawn**  **On** (*dd/Mmm/yyyy*) | **☐** | **☐** | **☐** | **☐** | **☐** | **☐** | **☐** |
|  | ____ / _____ / ________ | ____ / _____ / ________ | ____ / _____ / ________ | ____ / _____ / ________ | ____ / _____ / ________ | ____ / _____ / ________ | ____ / _____ / ________ |
| **Drug interrupted** | ☐ | ☐ | ☐ | ☐ | ☐ | **☐** | **☐** |
| **From** (*dd/Mmm/yyyy*) | ____ / _____ / ________ | ____ / _____ / ________ | ____ / _____ / ________ | ____ / _____ / ________ | ____ / _____ / ________ | ____ / _____ / ________ | ____ / _____ / ________ |
| **To** (*dd/Mmm/yyyy*) | ____ / _____ / ________ | ____ / _____ / ________ | ____ / _____ / ________ | ____ / _____ / ________ | ____ / _____ / ________ | ____ / _____ / ________ | ____ / _____ / ________ |
| **Not applicable** | **☐** | **☐** | **☐** | **☐** | **☐** | **☐** | **☐** |
| **Event diminished after drug stopped/dose reduced?** | **Yes ☐ / No ☐ / N/A ☐** | **Yes ☐ / No ☐ / N/A ☐** | **Yes ☐ / No ☐ / N/A ☐** | **Yes ☐ / No ☐ / N/A ☐** | **Yes ☐ / No ☐ / N/A ☐** | **Yes ☐ / No ☐ / N/A ☐** | **Yes ☐ / No ☐ / N/A ☐** |
| **Event reappeared after drug/dose reintroduction?** | **Yes ☐ / No ☐ / N/A ☐** | **Yes ☐ / No ☐ / N/A ☐** | **Yes ☐ / No ☐ / N/A ☐** | **Yes ☐ / No ☐ / N/A ☐** | **Yes ☐ / No ☐ / N/A ☐** | **Yes ☐ / No ☐ / N/A ☐** | **Yes ☐ / No ☐ / N/A ☐** |

| **Causality assessment** | | SAE 1 | | | | | | | SAE 2 | | | | | | | SAE 3 | | | | | | |
| --- | --- | --- | --- | --- | --- | --- | --- | --- | --- | --- | --- | --- | --- | --- | --- | --- | --- | --- | --- | --- | --- | --- |
| **Related** **to Drug No.** | | 1 | 2 | 3 | 4 | 5 | 6 | 7 | 1 | 2 | 3 | 4 | 5 | 6 | 7 | 1 | 2 | 3 | 4 | 5 | 6 | 7 |
|  |  | ☐ | ☐ | ☐ | ☐ | ☐ | ☐ | ☐ | ☐ | ☐ | ☐ | ☐ | ☐ | ☐ | ☐ | ☐ | ☐ | ☐ | ☐ | ☐ | ☐ | ☐ |
|  | **Other drugs, specif**y: | …………………………….……………………………………………. | | | | | | | …………………………….…………………………………………….. | | | | | | | …………………………….…………………………………………… | | | | | | |
| **Not related to Drug No.** | | 1 | 2 | 3 | 4 | 5 | 6 | 7 | 1 | 2 | 3 | 4 | 5 | 6 | 7 | 1 | 2 | 3 | 4 | 5 | 6 | 7 |
|  |  | ☐ | ☐ | ☐ | ☐ | ☐ | ☐ | ☐ | ☐ | ☐ | ☐ | ☐ | ☐ | ☐ | ☐ | ☐ | ☐ | ☐ | ☐ | ☐ | ☐ | ☐ |
|  | **Other drugs, specif**y: | …………………………….……………………………………………. | | | | | | | …………………………….……………………………………………. | | | | | | | …………………………….…………………………………………… | | | | | | |
| Other causal factors (incl. med.history, procedure, etc.) | | …………………………….……………………………………………. | | | | | | | …………………………….……………………………………………. | | | | | | | …………………………….…………………………………………… | | | | | | |

| **Event description**  Provide a clear description of the sequence of events, diagnosis, relevant investigation results (ECG, CT scan, etc.), corrective treatments, evolution. |  |
| --- | --- |

| Relevant laboratory tests | | | | | | | | |
| --- | --- | --- | --- | --- | --- | --- | --- | --- |
| **Test** | | Date (*dd/Mmm/yyyy*) | | Result (unit) | | | Reference range | |
|  | | ____ / _____ / ________ | |  | | |  | |
|  | | ____ / _____ / ________ | |  | | |  | |
|  | | ____ / _____ / ________ | |  | | |  | |
|  | | ____ / _____ / ________ | |  | | |  | |
| Concomitant medications | | | | | | | | |
| **Drug name (INN)** | **Daily dose and route** | | **Indication** | | **Treatment start date** (*dd/Mmm/yyyy*) | **Treatment stop date** (*dd/Mmm/yyyy*) | | **Continued** |
|  |  | |  | | **____ / _____ / ________** | **____ / _____ / ________** | | □ Yes □ No |
|  |  | |  | | **____ / _____ / ________** | **____ / _____ / ________** | | □ Yes □ No |
|  |  | |  | | **____ / _____ / ________** | **____ / _____ / ________** | | □ Yes □ No |
|  |  | |  | | **____ / _____ / ________** | **____ / _____ / ________** | | □ Yes □ No |
|  |  | |  | | **____ / _____ / ________** | **____ / _____ / ________** | | □ Yes □ No |

| **Relevant medical history**  Indicate relevant medical history, including prior diagnoses, past laboratory investigations, X-ray, ECG prior to treatment, previous procedures, and relevant past drugs. |  |
| --- | --- |

| **Reporter** | | | | |
| --- | --- | --- | --- | --- |
| **Name of reporter:** | **Role in trial/program:** | **Date of event’s awareness:**  ***ALL SAEs to be reported within 24 hrs of awareness***  **____ / _____ / ________** | **Address:**  **Email:**  **Phone:** | **Date and signature:**  **____ / _____ / ________** |

| **Further information on this SAE expected?** | **Yes ☐ No ☐**  *If yes please send a follow-up report once new information is available* | **Any annex to this document?** (e.g. discharge summary, autopsy report, lab results) | **Yes ☐ No ☐**  *If yes, list the annexes:* |
| --- | --- | --- | --- |
